# Astrocytic determinant of the fate of long‐term memory

**DOI:** 10.1002/glia.24636

**Published:** 2024-11-04

**Authors:** Hiroki Yamao, Ko Matsui

**Affiliations:** ^1^ Super‐network Brain Physiology, Graduate School of Life Sciences Tohoku University Sendai Japan; ^2^ Super‐network Brain Physiology, Graduate School of Medicine Tohoku University Sendai Japan

**Keywords:** amygdala, Astrocytes, fear conditioning, memory, meta‐plasticity, optogenetics

## Abstract

While some vivid memories are unyielding and unforgettable, others fade with time. Astrocytes are recognized for their role in modulating the brain's environment and have recently been considered integral to the brain's information processing and memory formation. This suggests their potential roles in emotional perception and memory formation. In this study, we delve into the impact of amygdala astrocytes on fear behaviors and memory, employing astrocyte‐specific optogenetic manipulations in mice. Our findings reveal that astrocytic photoactivation with channelrhodopsin‐2 (ChR2) provokes aversive behavioral responses, while archaerhodopsin‐T (ArchT) photoactivation diminishes fear perception. ChR2 photoactivation amplifies fear perception and fear memory encoding but obstructs its consolidation. On the other hand, ArchT photoactivation inhibits memory formation during intense aversive stimuli, possibly due to weakened fear perception. However, it prevents the decay of remote fear memory over three weeks. Crucially, these memory effects were observed when optogenetic manipulations coincided with the aversive experience, indicating a deterministic role of astrocytic states at the exact moment of fear experiences in shaping long‐term memory. This research underscores the significant and multifaceted role of astrocytes in emotional perception, fear memory formation, and modulation, suggesting a sophisticated astrocyte‐neuron communication mechanism underlying basic emotional state transitions of information processing in the brain.

## Main Points

- Opposing astrocyte manipulations cause fear‐like states or reduce fear.
- Astrocytes, at the moment of an experience, determine the long‐term memory fate.
- Astro‐ChR2 suppresses memory consolidation, while Astro‐ArchT inhibits remote memory decay.

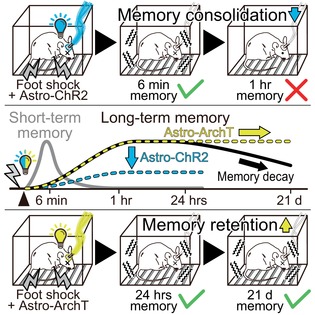

## 1 INTRODUCTION

Humans and animals make use of the repetitive nature of incidents in their surroundings and store the apparent correlative relationships in perceptions as memory and refer to them for adaptive behavior and survival. Multi‐phase memory formation has been well recognized, and short‐ and long‐term memory formation has generally been assumed to proceed serially. However, it is also possible that totally different cellular processes leading to short‐ and long‐term memory are initiated simultaneously but independently at the very moment of the memorable experience. The difference between short‐ and long‐term memory could be in the delay before the memory can manifest. It is thus possible that the fate of memory, that is, whether the memory will be stored or discarded in the long run, is already determined by the initial cellular parameters at the time of the experience. If multi‐phase memories are formed through such parallel processes, specific perturbation of the neuronal circuit may create enough divergence to reveal the presence of these processes.

Here, we used the classical fear conditioning paradigm as a model to dissect the multi‐phase memory formation process. Fear responses are mediated by complex neural mechanisms and structures, the most central of which is the amygdala (Ledoux, 2000). One of the nuclei within this structure, the basolateral amygdala (BLA), plays a key role in the processing of emotional reactions, particularly those related to fear (Jhang et al., 2018; Wei et al., 2015). Furthermore, extensive studies have highlighted the BLA as a critical site for fear memory, where it functions in the acquisition, storage, and recall (Kim et al., 2013; Kim & Cho, 2020; Kitamura et al., 2017; Nonaka et al., 2014) of fear memories.

As a means to perturb the memory formation in anterior BLA (aBLA) circuits, we employed astrocyte specific optogenetics. Astrocytes have been increasingly acknowledged as active contributors to neuronal excitability (Araki et al., 2024; Karus et al., 2015; Lezmy et al., 2021; Nagai et al., 2019; Onodera et al., 2021; Pabst et al., 2016; Saab et al., 2012; Wahis et al., 2021), synaptic transmission (Beppu et al., 2021; Bernardinelli et al., 2014; Chever et al., 2014; Di Castro et al., 2011; Matsui & Jahr, 2003; Panatier et al., 2011; Perea & Araque, 2007; Sasaki et al., 2012; Untiet et al., 2023) and plasticity (Adamsky et al., 2018; Henneberger et al., 2020; Iwai et al., 2021; Kanaya et al., 2023; Kol et al., 2020; Navarrete et al., 2012; Zhou et al., 2021), as well as the construction and refinement of neural circuits (Asrican et al., 2020; Lee et al., 2021; Morizawa et al., 2022), controlling the brain's operative state. Astrocytic regulation of fear and fear memory has been suggested (Badia‐Soteras et al., 2023; Iwai et al., 2021; Li et al., 2020; Martin‐Fernandez et al., 2017; Suthard et al., 2023). However, the extent of their involvement in regulating emotional states and associated memory formation processes in animals is not yet fully understood. We used channelrhodopsin‐2 (ChR2) and archaerhodopsin‐T (ArchT) specifically expressed in astrocytes and examined their effects on fear‐related behaviors and fear memory formation in mice. The rapid and transient nature of optogenetic manipulations ideally suits the investigation of transitional astrocytic mechanisms that underpin the acute regulation of animals' emotional states and memory formation processes.

The discoveries presented in this research unveil fresh perspectives into the intricate dynamics of astrocyte‐neuron interactions. Aberrant fear responses and fear memory formation are core features of numerous psychiatric disorders, such as post‐traumatic stress disorder. Understanding the intricate mechanisms underlying fear perception and fear memory formation is thus not only of fundamental interest to neuroscience but also holds the key to developing more effective treatments for fear‐related disorders. This research underscores the promise of astrocytic manipulation as an innovative frontier in devising therapeutic strategies for disorders related to fear.

## 2 MATERIALS AND METHODS

### 2.1 Animals

This study was conducted in accordance with the recommendations of the Regulations for Animal Experiments and Related Activities at Tohoku University, and all experimental procedures were approved by the Institutional Animal Care and Use Committee of Tohoku University (2019LsA‐017). We took every effort to minimize animal suffering and reduce the number of animals used. During stereotaxic surgery, all mice were regularly checked for toe‐pinch pain responses to ensure they remained unconscious and did not feel pain. All mice were handled before the experiments (see Section 2.4 for further details). To mitigate the fear induced by human contact and reduce stress, the mice were cup‐handled and allowed to move freely on the experimenter's palm during transportation. After the experiments, the mice were sacrificed as quickly as possible using an overdose of isoflurane to prevent any residual stress (ensuring a peaceful process). The mice were then perfusion‐fixed, or whole‐head fixed in 4% Paraformaldehyde Phosphate Buffer Solution for the analysis of fiber insertion sites (see Section 2.2 for further details).

Mlc1‐tTA mice were generated using BAC transgenesis (Tanaka et al., 2010). In tetO‐ChR2(C128S)‐EYFP mice, the transgene was inserted into the β‐actin locus (Sasaki et al., 2012; Tanaka et al., 2012). In tetO‐ArchT‐EGFP mice, the transgene was integrated into the β‐actin gene through BAC transgenesis (Beppu et al., 2014). The tetO‐E^2^GFP mice were generated as previously described (Ikoma, Sasaki, & Matsui, 2023; Onodera et al., 2021; Tan et al., 2024). In the pH sensor mice, a genetic pH sensor (E^2^GFP; Bizzarri et al., 2006) was conjugated with a membrane‐tethering domain, a lymphocyte‐specific membrane‐spanning tyrosine kinase (Lck), to generate Lck‐E^2^GFP to monitor pH changes, especially in the submembrane domain; a similar strategy to the Lck‐GCaMP3 for Ca^2+^ (Shigetomi et al., 2010). All mice used in this study were male. The bigenic Mlc1‐tTA::tetO‐ArchT mice, Mlc1‐tTA::tetO‐ChR2 mice, and Mlc1‐tTA monogenic mice were used at 12–23 week old (*n* = 54), 10–24 week old (*n* = 71), and 9–23 week old (*n* = 21), respectively, at the time of conditioning. The mice were maintained under a 12:12 light–dark cycle, with ad libitum access to water and dry food. The behavioral experiments were conducted during the light cycle. Prior to surgical procedures, the mice were housed in groups (no more than six per cage), and after surgery, they were housed individually.

### 2.2 Optical fiber insertion to the brain by stereotaxic surgery

The implanted glass optical fibers (400 μm in diameter with an additional 20 μm of cladding, NA = 0.39) (FT400UMT, Thorlabs, USA) were positioned with their light‐emitting tips directly above the anterior basolateral amygdala (aBLA). aBLA plays a crucial role in the expression of behavior and memory evoked by fear. However, the activity in the posterior part of the BLA leads to the alleviation of anxiety and the formation of hopeful memories (Pi et al., 2020; Yang & Wang, 2017). Therefore, in this study, the optical fibers were positioned to allow light illumination in the anterior part of the BLA. The optical fibers were cut and polished perpendicularly to the longitudinal axis and subsequently cleaned in advance. They were attached to ceramic ferrules (2.5 mm in diameter, 10.5 mm in length) (CF440‐10, Thorlabs, USA) using epoxy resin, which enabled fiber‐to‐fiber connections.

For the stereotaxic surgeries, mice were anesthetized using isoflurane (induced at ~4%, maintained at ~2%) administered via a vaporizer (Univentor 400; Univentor Limited, Sweden). Vaseline (nacalai tesque, Japan) was applied over the eyes to prevent them from drying. The mice were secured to a stereotaxic frame using ear bars (Narishige, Japan), and a scalp incision was made along the midline of the head, revealing the skull. Adjustments were made so that the height difference between bregma and lambda was less than 0.15 mm. For the optogenetics experiments, bilateral holes were drilled into the skull, 1.40 mm posterior and 3.05 mm laterally from bregma using an electric drill (osada success 40, OSADA ELECTRIC CO., Japan) and a precision surgical tool (10063‐15 Micro point, Fine Science Tools Inc., Canada). For the fiber photometry experiments, unilateral holes on the left hemisphere were drilled into the skull 1.45 mm posterior and 3.10 mm laterally from bregma. The optical fibers were vertically inserted into these holes and positioned 3.50 mm ventrally for the optogenetics experiments, and 4.10 mm ventrally for the fiber photometry experiments from the surface of the brain. The optical fibers were secured in their position by fixing them to the skulls using dental adhesive resin cement (Super‐Bond C&B, SUN MWDICAL CO., Japan). Subsequently, the surrounding area was coated with dental cement (REPAIRSIN, GC Corporation, Japan), which was mixed with charcoal to create a black seal in order to prevent light leakage into the brain from sources other than the fibers. After these procedures, the fibers and ferrules protruded approximately 14 mm from the skull, thereby allowing for connection with other optical fibers.

**FIGURE 1:**
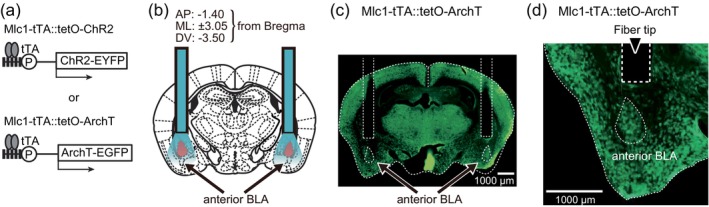
Astrocyte specific optogenetic manipulation in the amygdala. (a) Schematic of tTA‐mediated ChR2 and ArchT expression. (b) Schematic of bilateral optical fiber insertion above the anterior basolateral amygdala (aBLA). (c) Tracing the placement of optical fibers in the brain of the Mlc1‐tTA::tetO‐ArchT mouse in 100 μm thick coronal brain sections. (d) Enlarged view of the optical fiber trace alongside ArchT expression in the aBLA.

Stereotaxic surgeries were done with mice older than 8 weeks, and the behavioral experiments were conducted following a recovery period of at least 7 days post‐surgery. The optical fiber implantation site was verified after the completion of the behavioral experiments. The mice's brains were extracted from the skull following perfusion fixation or by whole‐head fixation in 4%‐Paraformaldehyde Phosphate Buffer Solution (nacalai tesque, Japan). Brain sections of 50–100 μm were prepared using a vibratome (Leica VT1000S, Leica Biosystems, Germany), and were mounted onto slide glasses. Mice with excessive displacement of the location of the optical fibers were excluded from the behavioral experiment analyses. An inverted fluorescence microscope (ZEISS Axio Imager 2, Carl Zeiss AG, Germany) was used to create images presented in Figure 1c,d.

### 2.3 Intracranial optical stimulation via optical fibers

For *in vivo* photoactivation in freely moving mice, an LED light source (Lumencore SPECTRA3, Lumencore, USA) was used. The light source was connected to a glass optical fiber (1500 μm diameter, NA = 0.39) (FT1500MT, Thorlabs, USA) via an FC connector. The light from the glass optical fiber was split into a pair of plastic optical fibers (500 μm diameter, NA = 0.5) (Luminous, Asahi Kasei, Japan). The two plastic optical fibers were connected to each of the glass optical fibers implanted bilaterally into the mice's brains via ferule‐ferule connections using split sleeves (ADAF1‐5, Thorlabs, USA). The plastic optical fibers were chosen for their flexibility, which ensured unrestricted movement of the mice within the experimental environment.

For ChR2 (C128S) photoactivation, a sequence of 500 ms blue light pulse (475/28 nm; 1.8–2.5 mW at fiber tip) immediately followed by 200 ms yellow light pulse (575/25 nm; 1.8–2.5 mW at fiber tip) was applied. For ArchT photoactivation, a 20‐s yellow light pulse (3.8–4.5 mW at fiber tips) was applied. A pulse generator (Master‐8, AMPI, Israel) was used to trigger the LED light source. Typically, during the fear conditioning session, immediately following the electrical foot shock, the light impulse was delivered, and, with a start‐to‐start interval of 120 s, two more light impulses were subsequently delivered. Importantly, the light impulses were always delivered after the electrical foot shock, which ensured that the sensation of pain by the foot shock would not be interfered with by the optogenetic manipulation. In the delayed photostimulation experiments, three light impulses with 120‐s intervals were delivered 33 min after the conclusion of the conditioning session.

In control mice with no photostimulation, light impulses were still delivered to the optical fibers, but three layers of aluminum foil sandwiched between the ferrule‐ferrule connection of the intracranial glass optic fiber and the relay plastic optical fiber prevented the light from reaching the brain. As translucent relay plastic optical fibers were used, significant visual light was always side‐emitted from the relay fibers when light impulses were delivered. Such side‐emitting light could be visualized by the mice and may affect their behavior. Thus, the above configuration was used in control mice to ensure consistent visual input to the mice as in the real brain photostimulation sessions.

### 2.4 Behavioral experiment apparatus

Behavioral experiments were conducted using freely moving mice, mainly within a soundproof experimental box (O'HARA & CO., Japan). The experimental setup was situated in a small, isolated room outfitted with an adjustable ventilation fan, which produced a characteristic rotational noise. Video recordings were obtained during these experiments for quantitative behavioral analysis. To deliver aversive electrical foot shocks, the mice were introduced into a square, translucent acrylic chamber featuring a floor made of metal grids with a floor area of 33 cm × 25 cm ([context A]) (O'HARA & CO., Japan), within the soundproof experimental box. These grids were connected to a shock generator (O'HARA & CO., Japan), which produced 125 Hz scramble foot shocks. The current was applied to two adjacent grids out of eight sequentially arranged grids at a time, with the targeted grids changing every 1 ms to generate a scramble shock. The current applied through the grids was determined using a 75 kΩ dummy load circuit within the shock generator prior to scrambling. [Context A] was employed for the habituation, conditioning, and contextual fear test sessions. Optical fibers were also introduced from outside the soundproof experimental box into this setup to enable *in vivo* optical stimulation during the experiments. [Context A] was thoroughly cleaned with 70% ethanol immediately prior to the introduction of the mice in each experimental session. Furthermore, a 60 dB white noise was presented within the soundproof experimental box throughout the duration of the experiments. The brightness within [context A] was set to 100 lux for all experiments.

During the cued fear tests, a different triangular opaque chamber with a floor area of an equilateral triangle with sides of 32 cm [context B], was placed and used inside the soundproof experimental box as [context A]. The floor of [context B] was plain rough‐textured plastic. [Context B] was thoroughly cleaned with a diluted sodium hypochlorite bleach solution (Kao Corporation, Japan) immediately prior to the introduction of the mice in each experimental session to contrast the odor of [context A]. Furthermore, the white noise presented within the soundproof experimental box was reduced to 50 dB during the cued fear tests. The brightness within [context B] was also uniformly set to 100 lux for all experiments, and video recordings were obtained. The ventilation fan of the room was set to a significantly lower speed during the cued fear tests, causing it to produce a much lower amplitude and low frequency noise compared to during the experiments in [context A].

For the delayed ChR2 photoactivation experiments, the mice were transferred to a separate, plastic, translucent chamber with a floor area of 17.5 cm × 17.5 cm [context C], located within a different experimental box from [context A], but within the same room. The floor of [context C] was covered with paper bedding chips (Alpha‐dri, Shepherd Specialty Papers, USA). Plastic optical fibers were also introduced into this experimental box to enable *in vivo* optical stimulation.

For the experiments conducted in [context A] and [context C], the experimenter wore a cloth gown, along with a face mask, hair cap, and large nitrile gloves. For the experiments conducted in [context B], the experimenter wore a vinyl gown, along with a face mask, hair cap, and smaller latex gloves, in order to create a differentiation from the experiments conducted in [context A] and [context C].

### 2.5 Fear conditioning paradigm

The contextual and cued fear conditioning paradigm was utilized to evaluate fear memory in mice, adapted and modified from the methodology of Shoji et al. (Shoji et al., 2014).

Before the conditioning sessions, the mice were acclimatized to [context A] in habituation sessions over two consecutive days, where they were allowed to freely explore the environment for 8 min each day. This served to mitigate the high motility usually exhibited by mice in a novel environment due to their exploration drive. This ensured a more consistent measure of their behaviors across different sessions. On the third day, the mice were reintroduced into [context A], and their pre‐conditioning behavior (day prior tests) was recorded during an 8‐min session. On the fourth day, conditioning took place, where the mice were subjected to electrical foot shocks. Following the conditioning sessions, 8‐min long cued and contextual fear tests were conducted after a designated interval to assess the mice's fear memories.

For all sessions conducted in [context A], the mice were transported to the experimental room while still in their home cages. Following this, they were carefully taken out of their cages via cup handling and allowed to move freely on the palm of the experimenter's hand for 3 min. This handling procedure was implemented to mitigate the fear induced by human contact and to acclimate the mice to human interaction, as well as to standardize the level of awakeness and alertness of the mice across sessions. After the relay plastic optical fibers were connected to the intracranial optical fibers of each mouse, the mice were then introduced into the experimental setup. The fiber connection procedure was not applicable for experiments that were used to demonstrate the transition of fear memories over time using ChR2 non‐expressing Mlc1‐tTA mice (Figure S3) and the delayed ChR2 photoactivation experiments. The experiments for each mouse were carried out approximately at the same time of the day, with a maximum of a 2‐h difference between any of the session execution times. This applies to all habituation and test sessions.

During the conditioning sessions, the mice were either subjected to 0.3 mA, 2‐s‐long, or a 0.7 mA, 4‐s‐long foot shocks. In the delayed ChR2 photoactivation experiments, the mice received three 0.3 mA, 2‐s‐long foot shocks at 2.5, 4.5, and 6.5 min into the 8‐min conditioning sessions. For all other experiments, the mice received a single foot shock 2.5 min into the 8‐min conditioning sessions. For the ChR2 experiments, except for the delayed ChR2 photoactivation experiments, the mice received a 0.7 mA, 4‐s‐long foot shock. For the ArchT experiments, the mice received either a 0.3 mA, 2‐s‐long or a 0.7 mA, 4‐s‐long foot shock. In cases where the mice were later subjected to cued fear tests, a 30‐s‐long, 10 kHz tone was also presented, preceding and co‐terminating with the foot shocks. For the delayed ChR2 photoactivations, the mice were immediately moved to context C, where they were connected to the relay plastic optical fibers and were allowed to move freely. Three pulses of photostimulations were delivered starting 33 min after the end of the conditioning session, each separated by 120‐s intervals from the start of each pulse. The activity of the mice in [context C] was monitored via a live camera system, and the photostimulations were confirmed through this observation.

For the contextual fear tests, the mice were evaluated at one or more time points: 6 min, 52 min, 24 ± 2 h, or 21 days after the conditioning session. For these sessions, the mice were handled and, in most cases, connected to the relay plastic optical fiber (except for the experiments that were used to demonstrate the transition of fear memories over time using ChR2 non‐expressing Mlc1‐tTA mice and the delayed ChR2 photoactivation experiments), then reintroduced to [context A]. For the mice that did not undergo contextual fear tests on the same day as the conditioning sessions (6 or 52 min tests), the cued fear tests were also conducted using the same mice (Figure S4). For those that were subjected to contextual fear tests on the same day (6 or 52 min tests) as the conditioning sessions, only the contextual fear tests were conducted.

For the mice that underwent the cued fear tests, they were invariably subjected to contextual fear tests as well. The cued fear tests were conducted 4 ± 0.5 h before the contextual fear tests, either only on the following day or also 21 days after the conditioning sessions. For the cued fear test, the mice were transported to the experimental room while still in their home cages. However, the mice were taken out from their home cages in a different location than the one used during the experimental procedures of the contextual fear tests. The 3‐min handling procedure was not implemented, and no relay plastic optical fibers were connected to the mice during the sessions. Out of the 8‐min session, the initial 2 min were not used for analysis, serving as an adaptation period since the mice tended to show especially high motility during this period, likely due to their exploration drive in a new environment. The 10 kHz tone, which was also presented during conditioning, was played for 3 min starting from the 5‐min mark of the 8‐min sessions. The freezing levels during a 3‐min period before and during the presentation of the 10 kHz tone was compared.

Between individual sessions, the mice were returned to their home cages in the animal facility in the lab. The timing for camera imaging, foot shocks, 10 kHz tones, and light exposures were all managed through the Interface equipment and Time FZ1 software from O'HARA & CO., Japan.

### 2.6 Video analysis of mice behavior

The animal's position, motility, and the presence or absence of freezing behavior and tail rattling behavior were assessed. The detection of freezing behavior was automatically evaluated using FIJI software (NIH, USA) and Python (3.8.3) to ensure the exclusion of any human bias and inaccuracies. For the data processing in Python, the standard libraries and Pandas (1.0.5), Numpy (1.18.5), Scipy (1.5.0) were used.

Video recordings in [context A] were acquired using an overhead CCD camera (WAT‐902B, Watec Co., Japan), while recordings in [context C] were acquired using an overhead CMOS camera (BSW200MBK, BUFFALO INC., Japan). In [context A], a 312 × 239‐pixel image was acquired in black and white at a rate of 30 frames per second, while in [context C] a 290 × 258‐pixel image was acquired in full color, respectively capturing the entire base area of the chamber with a slight additional margin. The video captured in [context C] was converted to black and white for analysis. Given the dark fur color of the mice, the background of the experimental setups was adjusted to a light tone, ensuring the mice were easily distinguishable from the background. The silhouettes of the mice were obtained through binary segmentation of the images captured from above the mice. The position of the animal during the experiments was determined using an ImageJ (NIH, USA) macro program. Initially, the pixels of the mice silhouettes were extracted by setting a threshold in the image brightness and converting the images to binary. Next, a median filter was applied to each image in order to remove the silhouette of the mice's feces and tails and impulse noise. This step was necessary because the position and orientation of the tail, as well as the location of any feces, could significantly affect the calculation of the center‐of‐mass of the silhouette, which was used to define the position of the mice in the experimental environment. Furthermore, the distance traveled by the mice was calculated as the difference in the center‐of‐mass position between frames using an algorithm developed in Python.

Subsequently, the freezing behaviors were assessed using the FIJI software and a Python algorithm. Freezing behavior was defined as any period during which the mouse's motion fell below a certain threshold for more than 2 s (60 consecutive frames). This was calculated by determining the number of pixels showing differences between consecutive frames (computed via the XOR operation in FIJI). During freezing behaviors, which are characterized by the absence of any movement apart from respiration, mice become noticeably still. As a result, the difference between continuous video frames capturing the mice's movement dramatically decreases compared to when the mice are in motion. Any periods in which these values fell below the set threshold for more than 2 s (60 consecutive frames) were computed as instances of freezing behavior. A median filter was applied in advance to remove impulse noise before calculating these values. This threshold for immobility determination was carefully adjusted to ensure a substantial match between instances identified as freezing behaviors by the experimenter's manual assessment using the original video and those identified as freezing behaviors in the Python program's output. The plastic optical fibers that relayed inputs into the intracranial optic fibers were used without any opaque coating, remaining transparent, and therefore appeared bright on the camera, similar to the background, ensuring its movement did not influence the center‐of‐mass or difference between continuous frames. However, when the optical fiber passed between the mouse's body and the camera, it caused fluctuations in the center of mass and an increase in the difference between continuous frames, resulting in rare cases where accurate freezing behavior judgment could not be conducted.

The occurrence of tail rattling behavior was determined manually by the experimenters through assessments of the video footage.

### 2.7 Immunohistochemistry

Mice were deeply anesthetized with isoflurane, and perfusion fixated. 15 mL of Phosphate‐buffered saline (PBS), followed by 30 mL of 4% PFA in PBS was transcardially perfused. The brains were then removed and post‐fixed in 4% PFA at 4°C overnight, and cryoprotected in 30% sucrose. They were then embedded in OCT compounds (Tissue‐Tek, Sakura Finetek Japan, Japan) and were frozen to be cut into 30 μm thick coronal sections using a cryostat microtome (Leica, Germany). Free floating sections were washed in PBS, incubated for 1 h in blocking solution (3% normal goat serum, 0.3% Triton X‐100 in PBS), washed in PBS, and then incubated in the primary antibody solution at 4°C overnight. The primary antibody solution contained either rabit anti‐GFAP (1:1000; Abcam, United Kingdom), mouse anti‐NeuN (1:100; Millipore, United States), or mouse anti‐S100β (1:800; Sigma‐Aldrich, United States) in a 3% normal goat serum, 0.1% Triton X‐100 in PBS solution. After washing with PBS, the sections were incubated in the secondary antibody solution at room temperature for 2 h. The secondary antibody solution contained either goat anti‐rabbit, Alexa Fluor 647 (1:1000; Invitrogen, United States), or goat anti‐mouse, Alexa Fluor 647 (1:400; Invitrogen, United States) in a 3% normal goat serum, 0.1% Triton X‐100 in PBS solution. After a final wash with PBS, the sections were mounted on slides with Vectashield Mounting Medium (Vector Lab, United States), and examined using an inverted fluorescence microscope (ZEISS Axio Imager 2, Carl Zeiss AG, Germany) (Figure S1).

### 2.8 Fiber photometry

For *in vivo* fiber photometry recordings in freely moving mice, a custom‐built device with an optical block series (Hamamatsu Photonics, Japan) was used for optical stimulation and measurement of the fluorescence output. This device was equipped with two photomultiplier tubes (PMTs) (H10722–210, Hamamatsu Photonics, Japan) and connected to an LED light source (Lumencore SPECTRA3, Lumencore, USA) via glass optical fibers (400 μm in diameter with an additional 30 μm of cladding, NA = 0.37) (Doric, Canada). The Excitation light (475/28 nm) was passed through a 473/24 nm filter (Chroma, USA) and directed into the brain, while the returning emission light was passed through either a 510/20 or 540/40 nm filter (Chroma, USA) and detected by the two PMTs.

To connect the module to the mice's brains, the implanted glass optical fibers were linked to a relay glass optical fiber (400 μm in diameter with an additional 20 μm of cladding, NA = 0.39) (FT400UMT, Thorlabs, USA), covered by opaque sheathing. The connection was made using ferrule‐ferrule connectors with split sleeves (ADAF1‐5, Thorlabs, USA). This relay fiber was further connected to the module via an FC connector.

To account for signal changes caused by factors like blood volume fluctuations or motion artifacts, the pH measurements were taken using the differential between the pH‐sensitive fluorescence wavelength (540 nm) and the isosbestic fluorescence wavelength (510 nm) (Figure S9a,b). Before using the measured data, autofluorescence from the glass optical fibers and the fiber photometry module's optical path was subtracted from the measurements. The autofluorescence of the optical fibers was determined by covering the fiber tips with a black cap to block external light. The autofluorescence values measured over 2–3 min were averaged and uniformly subtracted from the fluorescence values collected from the mice. The measurements were then normalized using the values from the first 142 s after the introduction into [context A] (before the electric foot shocks) to observe the percentage changes in fluorescence from baseline to post‐shock. The isosbestic fluorescence change ratio was subtracted from the pH‐sensitive fluorescence change ratio to isolate the effects of astrocytic pH changes on E^2^GFP fluorescence.

For the electric shock experiments, the mice were connected to the fiber photometry module and placed in a cage [context C]. After 1 h, they were moved to [context A], where they received six electric foot shocks (0.9 mA for 8 s) at 2.5, 3.5, 4.5, 5.5, 6.5 and 7.5 min into the 8‐min conditioning sessions. Before these foot shock experiments, the mice were habituated to the same experimental contexts without shocks for three days. The third day of habituation, without foot shocks, was used as the control (Figure S9d).

### 2.9 Quantification and statistical analyses

Quantification and Statistical analyses were conducted using Python with Pandas (1.0.5), Numpy (1.18.5), Matplotlib (3.2.2), Seaborn (0.10.1), and Scipy (1.5.0). All data presented in this study represent the mean ± SEM of the entire dataset. P‐values for statistical analyses are reported to three decimal places in the figure legends. Specific details regarding the statistical tests, statistics, sample sizes, and p‐values are described in the figure legends. The statistical values and specific details of the tests used are also provided in a separate table (Table S1), where a minimum of three significant figures are reported, and values are rounded to the nearest decimal place.

## 3 RESULTS

### 3.1 Induction of fear by astrocytic ChR2 photoactivation in the amygdala

To understand the role of astrocytes in fear, channelrhodopsin‐2 (ChR2), specifically expressed in astrocytes, was photoactivated *in vivo*. Astrocyte specific expression was achieved using the *Mlc1* promoter, which drove tetracycline transactivator (tTA) expression in our transgenic mice. This line of mice was crossed with a knock‐in mouse with the tetO promoter driven C128S variant of ChR2 (Sasaki et al., 2012; Tanaka et al., 2012). In the bigenic mice with both Mlc1‐tTA and tetO‐ChR2 genes (Mlc1‐tTA::tetO‐ChR2), tTA binds to tetO leading to the expression of ChR2 specifically in astrocytes (Figure 1a). ChR2 is a light‐gated cation channel with high permeability to protons, and, in the C128S variant, blue light opens the channel, and yellow light illumination accelerates the channel closure (Berndt et al., 2009). The specificity of ChR2 expression to astrocytes in the amygdala was confirmed using immunohistochemistry (Figure S1a). A pair of optical fibers were inserted bilaterally with their light emitting tips immediately above the anterior basolateral amygdala (aBLA) (Figure 1b–d).

**FIGURE 2:**
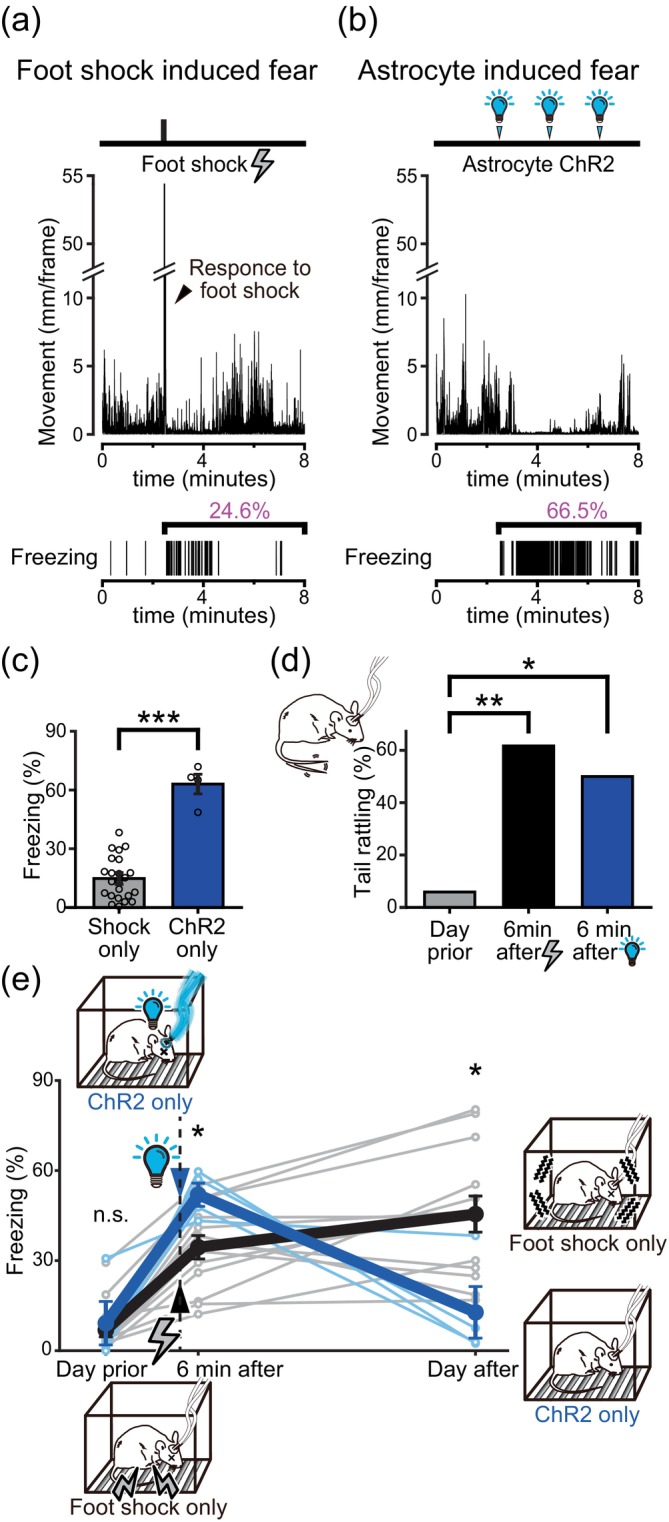
Astrocytic ChR2 photoactivation induced fear‐like behavior. (a,b) Examples of Mlc1‐tTA::tetO‐ChR2 mice reactions to a foot shock and astrocytic ChR2 photoactivation. Transition in movement (top) and freezing (bottom) during the session. Both foot shocks and ChR2 photoactivation induced an increase in freezing behavior. (c) Comparison of freezing levels (mean ± SEM) after a 0.7 mA 4‐s foot shock and astrocyte ChR2 photoactivation. The freezing percentage during the 5.5‐min period following the foot shock and during the 5.5‐min period when the mice were subjected to 3 astrocytic ChR2 photoactivations is shown (*p* = .000671 Welch's *t* test, ***<.001). *n* = 23 and 4 (foot shock group and ChR2 group). ChR2 photoactivation alone triggers substantial freezing behavior. (d) Comparison of the proportion of mice that exhibited tail rattling during the 8‐min sessions. Tail rattling on the day prior (*n* = 17) versus 6 min after the foot shock sessions (*n* = 13) (*p* = .000979 two‐proportion *Z* test, **<.005 with Holm correction), or versus 6 min after the astrocytic ChR2 photoactivation sessions (*n* = 4) (*p* = .0233 two‐proportion Z‐test, *<.05 with Holm correction). (e) Freezing levels during 8‐min sessions (mean ± SEM) tested on the previous day (*p* = .783 Welch's *t* test, >.05 with Holm correction), 6 min after the conditioning session (*p* = .0101 Welch's *t* test, *<.0125 with Holm correction), and on the following day (*p* = .0193 Welch's *t* test, *<.025 with Holm correction). Either a foot shock (*n* = 13) or astrocytic ChR2 photoactivation (*n* = 4) was delivered in the conditioning session (the same group of animals as in Figure 2d).

When a mouse is presented with an aversive electrical foot shock, a typical jumping behavior (i.e., escape response) followed by an increase in the freezing behavior is observed (Figure 2a). This freezing behavior is a defensive response frequently noted under stressful and fear‐inducing conditions (De Franceschi et al., 2016; Yilmaz & Meister, 2013); thus, the freezing level is often used as an indication of fear perception. Interestingly, astrocyte‐specific ChR2 photoactivation alone in the amygdala also induced a similar freezing behavior in the unrestrained mice (Figure 2b), and the level of freezing was even higher than that induced with an electrical foot shock of the intensity used (Figure 2c). It is possible that astrocytic ChR2 photoactivation ceased animal movement without producing fear. Therefore, as an alternative measure of fear and stress, tail rattling behavior was quantified (Borkar et al., 2020; Dorofeikova et al., 2023). The mice were subjected to a foot shock or astrocytic ChR2 photoactivation and were temporarily returned to their home cages. After a 6‐min interval, the mice were reintroduced to the experimental cage, where they previously experienced either the foot shock or the astrocytic ChR2 photoactivation. In both cases, an increase in freezing, as well as a higher occurrence of tail rattling behaviors, were observed (Figure 2d). This result suggests that the freezing observed in astrocytic ChR2 photoactivated mice is due to the induction of fear perception.

The fear produced by electrical foot shocks is often engraved robustly as a fear memory. Therefore, after 24 h spent in their home cages, freezing behavior is typically induced when the mice are reintroduced to the experimental cage with the same context (Figure 2e). The freezing levels are commonly regarded as an indicator of how well the memory is kept. When the mice were given astrocytic ChR2 photoactivation instead of the electrical foot shock, as introduced above, the freezing levels during the fear memory tests conducted 6 min after were significantly higher than those after the electrical foot shock. However, 24 h later, the freezing returned to the baseline level. This implies that, although astrocytic ChR2 photoactivation in the amygdala can induce strong fear‐like emotion, such fear experience fails to produce long‐term fear memory. It is possible that astrocytic ChR2 photoactivation inhibited long‐term memory formation.

**FIGURE 3:**
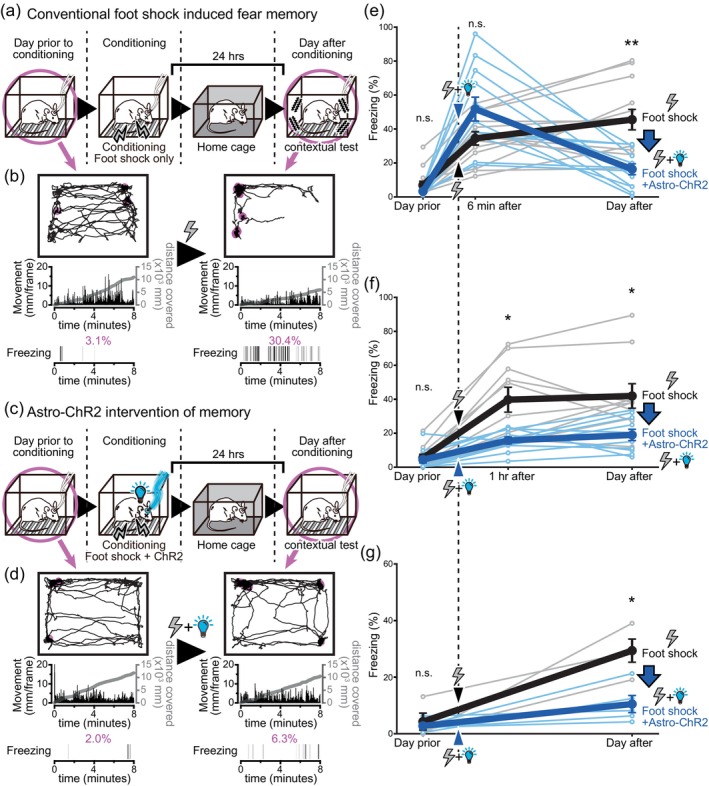
Astrocytic ChR2 photoactivation inhibits contextual fear memory formation. (a,c) Schematic of contextual fear conditioning with and without astrocytic ChR2 photoactivation during conditioning. (b,d) Examples of behaviors exhibited by Mlc1‐tTA::tetO‐ChR2 mice on the day before and after conditioning. The trajectory of mice during sessions is shown at the top, with magenta markers indicating locations where the mice displayed freezing. The transition in movement and the cumulative distance covered by the mice during the sessions are presented in the middle, and the freezing occurrences during the sessions is displayed at the bottom. Astrocytic ChR2 photoactivation disrupted formation of long‐term fear memory resulting from the electrical foot shock. (e) Freezing levels (mean ± SEM) when tested on the previous day (*p* = .151 Welch's *t* test >.05 with Holm correction), 6 min after (*p* = .0463 Welch's *t* test >.025 with Holm correction), and on the following day (*p* = .000439 Welch's *t* test **<.00334 with Holm correction) of the conditioning sessions. During conditioning, foot shock alone (*n* = 13, same sample set as used in Figure 2e) or foot shock in conjunction with astrocytic ChR2 photoactivation (*n* = 12) was delivered. Astrocytic ChR2 photoactivation did not interfere with short‐term memory formation, but the fear memory was dissipated on the following day. (f) Freezing levels (mean ± SEM) when tested on the previous day (*p* = .733 Welch's *t* test >.05 with Holm correction), approximately 1 h after (*p* = .00974 Welch's *t* test *<.0167 with Holm correction), and on the following day (*p* = .0128 Welch's *t* test *<.025 with Holm correction) of the conditioning sessions. Foot shock only group and foot shock+ChR2 group (*n* = 10 and 10, respectively). (g) Freezing levels (mean ± SEM) when tested exclusively on the previous day (*p* = .662 Welch's *t* test >.05 with Holm correction), and on the following day (*p* = .0103 Welch's *t* test *<.025 with Holm correction) of the conditioning sessions. Foot shock only group and foot shock+ChR2 group (*n* = 4 and 5, respectively).

### 3.2 Inhibition of memory formation by astrocytic ChR2 photoactivation

Next, the effect of astrocytic ChR2 photoactivation in the amygdala on the classic fear conditioning paradigm with an electrical foot shock was studied to evaluate whether astrocytic ChR2 photoactivation inhibits long‐term memory formation. An electrical foot shock induces a typical escape response. Astrocytic ChR2 photoactivation immediately following the foot shock failed to alter the escape response (Figure S2a,b). This suggests that the pain sensation caused by the foot shock is experienced similarly in the astrocytic ChR2 photoactivated mice. An immediate freezing response was observed in both conditions, after the electrical foot shock alone and when the foot shock was combined with astrocytic ChR2 photoactivation (Figure S2c). The freezing levels were higher with the concomitant stimulation. The tail rattling behavior was also often observed after the 6 min interval spent in their home cages and when the mice were reintroduced into the experimental cage (Figure S2d). These results indicate that fear experiences itself, and its short‐term (6 min) memory is not inhibited with astrocytic ChR2 photoactivation in the amygdala.

To gauge the extent of memory formation and retention at various points following fear conditioning with a foot shock, we conducted contextual fear tests at three distinct time intervals. After the foot shock was delivered, mice were reintroduced to the same context after a 6, 52‐min, or 24‐h interval. The freezing behavior during these sessions was monitored as an index of memory formation and retention. In these experiments, mice expressing Mlc1‐tTA without ChR2 expression were used. In these mice, increased freezing levels were observed in tests at all intervals (Figure S3). This shows that, with a single conditioning session with an electrical foot shock, fear memory is immediately formed and lasts for more than 24 h. When astrocytic ChR2 was photoactivated immediately after the foot shock, the freezing levels tested 24 h later was significantly reduced (Figure 3). In this group of mice, contextual fear memory experiments were combined with a cued fear memory experiment paradigm (Figure S4). The results of the cued fear memory tests will be introduced and discussed in the later sections. Conversely, the freezing levels tested 6 min after conditioning were similar to those in the group that received only the foot shock (Figure 3e). Nonetheless, the freezing on the day after conditioning was significantly reduced in astrocytic ChR2 photoactivated mice (Figure 3e). This attenuation of fear memory was also evident when tested 52 min post‐conditioning (Figure 3f).

**FIGURE 4:**
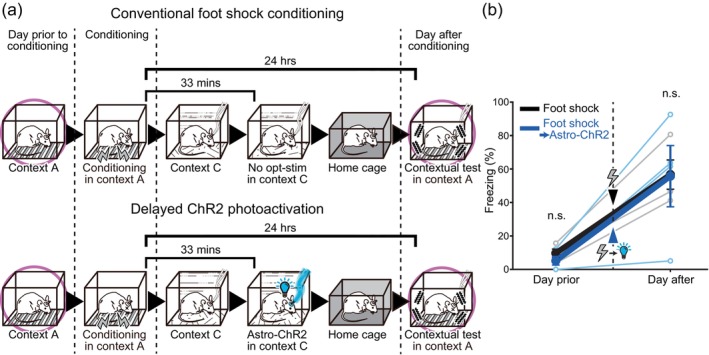
Delayed astrocytic ChR2 photoactivation failed to interfere with long‐term memory formation. (a) Schematic of contextual fear conditioning with and without delayed astrocytic ChR2 photoactivation. While only the contextual fear tests are depicted here, cued fear tests were also conducted with the same mice. (b) Freezing levels of Mlc1‐tTA::tetO‐ChR2 mice (mean ± SEM) when tested on the previous day (*p* = .314 Welch's *t* test >.025 with Holm correction) and on the following day (*p* = .965 Welch's t‐test >.05 with Holm correction) of the conditioning sessions. Foot shock only group and foot shock+delayed ChR2 group (*n* = 4 and 4, respectively). Delayed ChR2 photoactivation did not affect fear memory.

**FIGURE 5:**
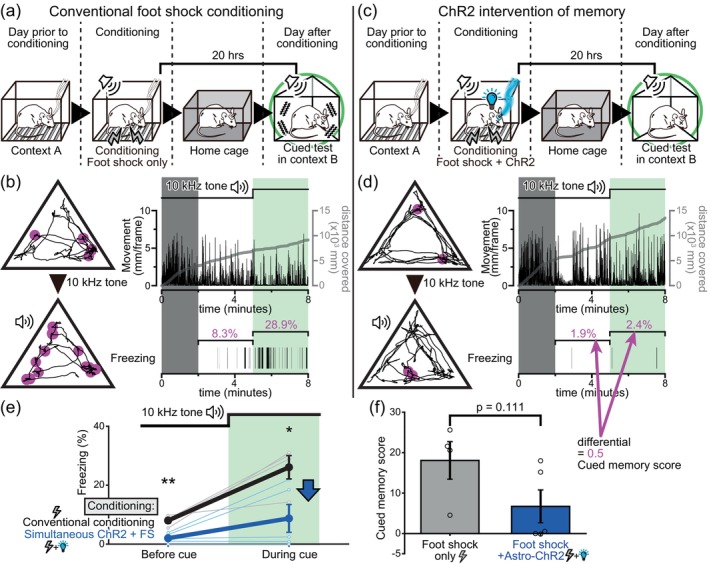
Astrocytic ChR2 photoactivation inhibits cued fear memory. (a,c) Schematic of cued fear conditioning with and without astrocytic ChR2 photoactivation during conditioning. The mice were introduced into a novel context [context B] on the following day of the conditioning sessions for the cued fear tests. During conditioning, foot shock alone or foot shock in conjunction with astrocytic ChR2 photoactivation was delivered to the mice. (b,d) Examples of behaviors exhibited by Mlc1‐tTA::tetO‐ChR2 mice during the cued fear tests. An initial adaptation period of 2 min was excluded. Freezing levels during the 3‐min period before and during the 10 kHz tone exposure were compared. Mice subjected to astrocytic ChR2 photoactivation during conditioning displayed low freezing levels both before and during the tone presentation. (e) Freezing levels (mean ± SEM) during the 3‐min period before (*p* = .00193 Welch's *t* test **<.005 with Holm correction) and during (*p* = .0259 Welch's *t* test *<.05 with Holm correction) the presentation of the 10 kHz tone is shown. Foot shock only group and foot shock+ChR2 group (*n* = 4 and 5, respectively; data from the same individual mice presented in Figure 3g). (f) Cued memory scores (Freezing% during tone − Freezing% before tone presentation) are shown. The data was taken from the same sample set as used in Figure 5e. *p* = .111 Welch's *t* test >.05. ChR2 photoactivation resulted in reduced cued memory scores, implying a disruption in the cued fear memory.

ChR2 photoactivation resulted in memory loss; however, it is possible that the memory may be suppressed during the 1 h to 24 h window post ChR2 photoactivation but may be recovered in the long run. Preliminary findings under varied conditions suggest that memory impaired by ChR2 photoactivation is unlikely to be naturally recovered over time. Specifically, the reemergence of fear memory after three weeks post‐conditioning with ChR2 photoactivation was never observed (data not shown).

Photostimulation via the optical fibers can generate heat in the illuminated tissue, potentially affecting neural activity (Owen et al., 2019). Mice expressing Mlc1‐tTA but lacking ChR2 expression were used to test whether heat generation alone has an impact on fear memory. Both a foot shock and optical stimulation to the amygdala were delivered in a group of mice, and the freezing levels were tested the following day. A robust increase in freezing was observed, which was comparable to the increase observed with foot shock alone (Figure S5a). This indicates that photostimulation, in the absence of ChR2 activation, does not affect the fear memory. It is also possible that two consecutive days of exposure to the experimental box induces an increase in freezing, even without the delivery of an aversive foot shock stimulus. This is highly unlikely as the mice had already been exposed to the experimental box for 3 consecutive days for habituation prior to the conditioning paradigm. Nonetheless, freezing levels in the experimental box were compared between the day prior and the day after the pseudo conditioning with no foot shock nor photostimulation (Figure S5b). Freezing levels were unchanged. This observation validates that a conditioning stimulus (i.e., the foot shock) is required for fear memory formation, expressed as an increase in freezing.

### 3.3 Limited time window of effective astrocytic memory intervention

The temporal time window for effective intervention of fear memory formation was investigated. After the foot shock conditioning in the experimental box [context A], the mice were transferred to a separate cage [context C]. In one group of mice, after a lapse of 33 min in [context C], astrocytic ChR2 was photoactivated. The mice were then transferred to their home cages and left undisturbed overnight. They were then tested for fear memory in [context A] the following day (Figure 4a). High freezing levels were observed the following day, and the astrocytic ChR2 photoactivation showed no effect when delivered with this sequence (Figure 4b). This result suggests that the interference of long‐term memory formation is only effective when the astrocytic ChR2 photoactivation is carried out within a narrow time window following the foot shock. Therefore, it seems that the astrocytic state at the precise moment of the aversive experience determines whether a memory will be consolidated and retained for a long time.

**FIGURE 6:**
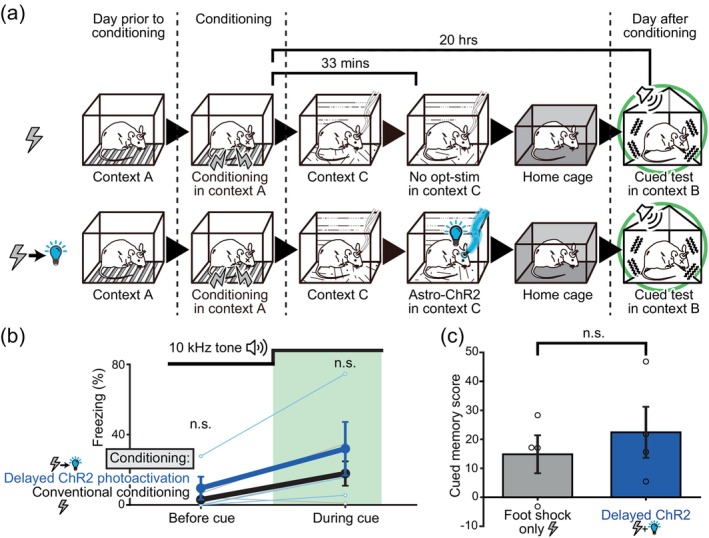
Delayed astrocytic ChR2 photoactivation failed to interfere with cued fear memory. (a) Schematic of cued fear conditioning with and without delayed astrocytic ChR2 photoactivation. (b) After conditioning, mice were subjected to either delayed astrocytic ChR2 photoactivation or not. Freezing levels (mean ± SEM) during the 3‐min period before (*p* = .397 Welch's *t* test >.025 with Holm correction) and during (*p* = .445 Welch's *t* test >.05 with Holm correction) the presentation of the 10 kHz tone is shown. Foot shock only group and foot shock+delayed ChR2 group (*n* = 4 and 4, respectively; data from the same individual mice presented in Figure 4b). (c) Cued memory scores (Freezing% during tone − Freezing% before tone presentation). The data is taken from the same sample set as used in Figure 6b. *p* = .517 Welch's *t* test >.05.

Consistent with the results in ChR2 photoactivation experiments without foot shocks, ChR2 photoactivation in [context C] also caused freezing behavior (Figure S6).

### 3.4 Astrocytic ChR2 photoactivation also inhibits cued fear memory

The effect of astrocytic ChR2 photoactivation on cued fear memory was also examined (Figure S4). Cued fear memory is also known to be dependent on synaptic plasticity in the amygdala (Meis et al., 2020; Sah et al., 2020). A 30‐s long 10 kHz tone was presented to the Mlc1‐tTA::tetO‐ChR2 mice placed in the conditioning experiment box, and a foot shock was delivered, which co‐terminated with the tone. The cued fear test was conducted approximately 20 h after the conditioning session. In this test, the mice were introduced to a novel environment (context B, triangular cage), and the 10 kHz tone was presented after 5 min. The first 2 min were excluded from the analysis, and the freezing during the 3‐min period preceding the tone (Before cue) and during the 3‐min period of the tone presentation (During cue) was compared (Figure 5a–d). In control mice, freezing levels “During cue” were higher than those “Before cue”, which shows that the mice recognized the cue and that a robust cued fear memory was formed during conditioning. When astrocytic ChR2 was photoactivated during conditioning, the freezing levels were significantly reduced during both the “Before cue” and “During cue” periods (Figure 5e). Significant reduction in freezing “During cue” shows that the cued fear memory formation is disrupted with the astrocytic ChR2 photoactivation. The significantly reduced freezing “Before cue” period could be due to the disrupted contextual fear memory. In control mice, the baseline freezing levels may be elevated during the cued test session in [context B] due to the similarities in the experimental procedures preceding the test, and the experimental environment. These processes are often referred to as memory generalization. In astrocytic ChR2 photoactivated mice, since contextual memory is also likely disrupted, baseline freezing levels “Before cue” may have become low. Out of the combined cued and generalized contextual fear memory effect, the freezing level explicitly induced by the cued fear memory was estimated by calculating the difference between the freezing levels “During cue” and “Before cue.” This “cued memory score” tended to be lower in the astrocytic ChR2 photoactivated mice compared to the foot shock only control mice, although the difference did not reach statistical significance (Figure 5f).

**FIGURE 7:**
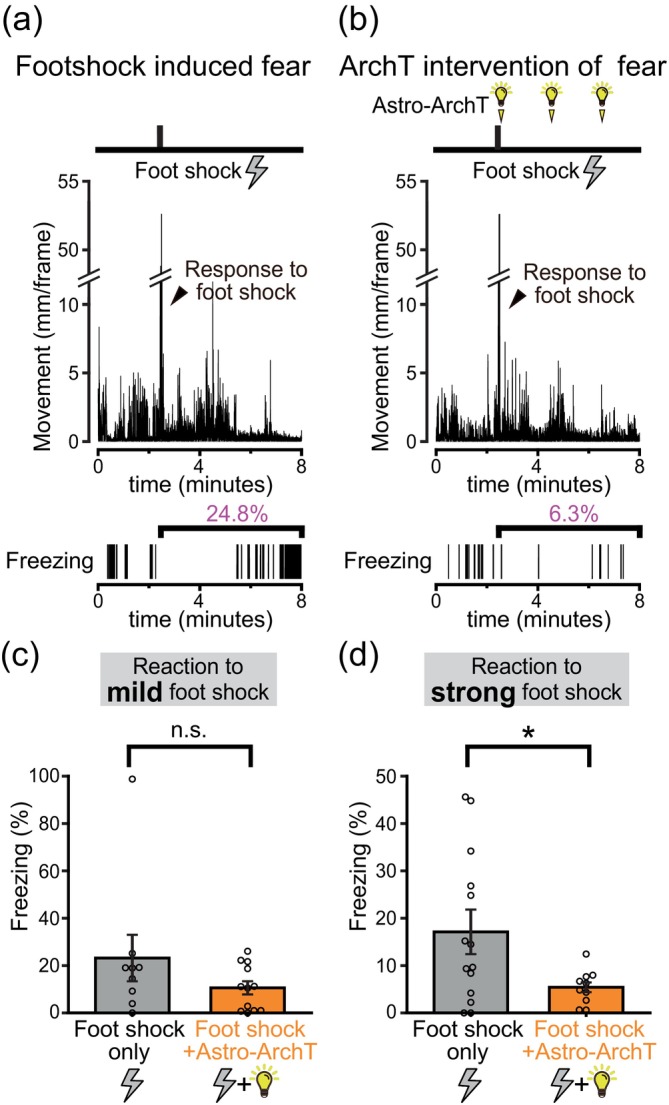
Astrocytic ArchT photoactivation reduces fear reaction. (a,b) Examples of mice reactions to a foot shock (0.7 mA for 4 s) and astrocytic ArchT photoactivation following the foot shock. A 30‐s long 10 kHz tone, co‐terminating with the foot shock, was also present during these sessions. Transition in movement (top) and freezing (bottom) during the session. Astrocytic ArchT photoactivation reduced the freezing behavior exhibited directly following a strong foot shock. (c,d) The mice were subjected to either a mild foot shock (0.3 mA for 2 s; (c)) or a strong foot shock (0.7 mA for 4 s; (d)). The freezing percentage during the 5.5‐min period following the foot shock is compared. In the mild foot shock group (c), no significant difference was observed in the freezing with foot shock only or foot shock+astrocytic ArchT photoactivation (*p* = 0.247 Welch's *t* test >.05, *n* = 9 and 12, respectively). In the strong foot shock group (d), compared to foot shock only, freezing was significantly less in foot shock+astrocytic ArchT photoactivation (*p* = .0368 Welch's *t* test *<.05, *n* = 14 and 10, respectively).

Photostimulation could produce heat or other unidentified influences on the brain tissue, possibly affecting cued fear memory. ChR2 non‐expressing Mlc1‐tTA transgenic mice were used, and the effect of photostimulation during conditioning on cued fear memory was studied. Similar levels of freezing “Before cue” and “During cue” and similar cued memory scores were observed between foot shock only mice and foot shock plus photostimulated mice (Figure S7a,b). This suggests that the photostimulation itself does not affect the formation or expression of cued fear memories. It is also possible that the 10 kHz tone alone could induce freezing behavior or fear memory formation. To test this possibility, mice that were not exposed to a foot shock or photostimulation during the conditioning session were prepared, and the cued fear test was conducted. Minimal freezing and cued memory scores were observed (Figure S7c,d). This shows that a conditioning stimulus (i.e., the foot shock) is required for the formation of cued fear memories.

Finally, the temporal window during which astrocytic ChR2 photoactivation could influence cued fear memory formation was investigated. Astrocytic ChR2 was photoactivated with a delay of 33 min from the foot shock conditioning session (Figure 6a). No effect of the astrocytic ChR2 photoactivation was observed on the cued fear memory tests (Figure 6). These results show that astrocytic ChR2 photoactivation is effective only when delivered within a narrow temporal window following the foot shock.

**FIGURE 8:**
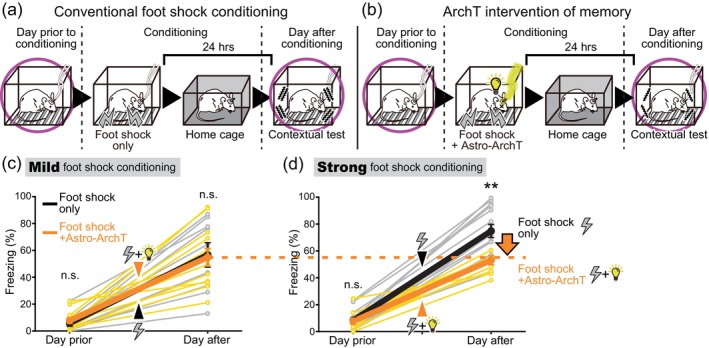
Astrocytic ArchT photoactivation inhibits intense memory formation. (a,b) Schematic of contextual fear conditioning with and without astrocytic ArchT photoactivation during conditioning. While only the contextual fear tests are depicted here, cued fear tests were also conducted with the same mice. (c) Freezing levels (mean ± SEM) when tested on the previous day (*p* = .538 Welch's *t* test >.025 with Holm correction), and on the following day (*p* = .877 Welch's *t* test >.05 with Holm correction) of the conditioning sessions. During conditioning, the mice were presented either a mild foot shock alone (0.3 mA for 2 s) or in conjunction with astrocytic ArchT photoactivation (*n* = 9 and 12, respectively; data from the same individual mice presented in Figure 7c. (d) Freezing levels (mean ± SEM) when tested on the previous day (*p* = .831 Welch's *t* test >.05 with Holm correction), and on the following day (*p* = .00145 Welch's *t* test **<.005 with Holm correction) of the conditioning sessions. During conditioning, the mice were presented either a strong foot shock alone (0.7 mA for 4 s) or in conjunction with astrocytic ArchT photoactivation (*n* = 14 and 10, respectively; data from the same individual mice presented in Figure 7d).

### 3.5 Astrocytic ArchT photoactivation reduces the immediate fear reaction

Archaerhodopsin‐T (ArchT) is a photoactivatable outward proton pump and the photoactivation of ArchT results in intracellular alkalinization (Beppu et al., 2014). Astrocyte specific expression of ArchT was achieved with Mlc1‐tTA::tetO‐ArchT mice, and the effect of amygdala illumination via a pair of optical fibers on fear memory was examined (Figure 1). The specificity of ArchT expression to astrocytes in the amygdala was confirmed using immunohistochemistry (Figure S1b). Astrocytic ArchT was photoactivated following an electrical foot shock, and the effect of the photoactivation on the immediate fear responses to the electrical foot shock was first examined (Figure 7a,b). In response to a foot shock alone, immediate freezing responses within the conditioning sessions were induced. Astrocytic ArchT photoactivation failed to produce a significant difference in the freezing levels when a mild foot shock (0.3 mA for 2 s) was used; however, a significant reduction in the freezing response was observed with astrocytic ArchT photoactivation when a strong foot shock (0.7 mA for 4 s) was used (Figure 7c,d). Therefore, astrocytic ArchT photoactivation is likely to be effective only against emotional responses elicited by excessive fear. Therefore, the selection and filtering of fear perception based on the significance or intensity of the fear experience was possibly perturbed by astrocytic ArchT photoactivation.

**FIGURE 9:**
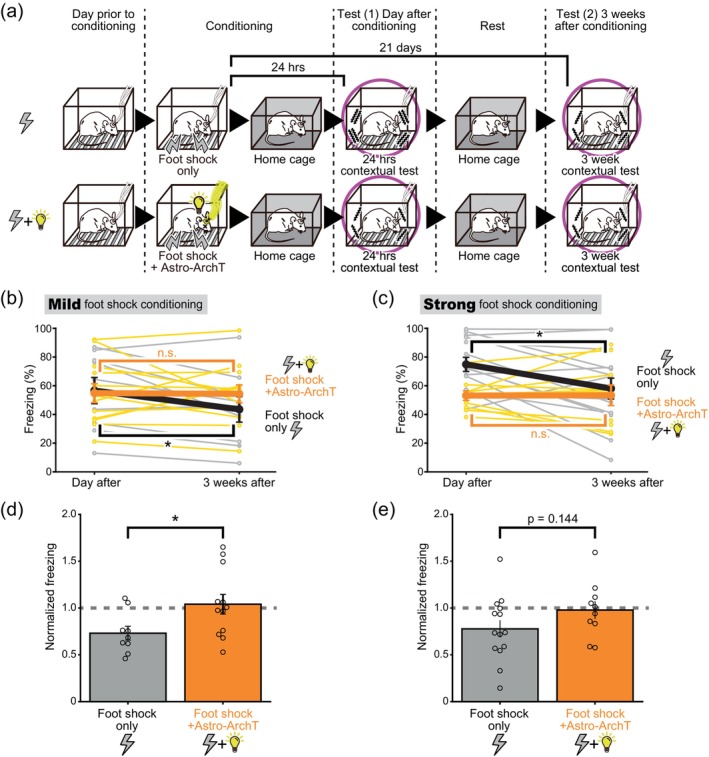
Astrocytic ArchT photoactivation inhibits remote memory decay. (a) Schematic of remote contextual fear tests with and without astrocytic ArchT photoactivation during conditioning. After the contextual fear test conducted on the day following conditioning, the mice were tested again 21 days post‐conditioning. While only the contextual fear tests are depicted here, cued fear tests were also conducted with the same mice. (b,c) Freezing levels (mean ± SEM) when tested on the following day, and 21 days after the conditioning sessions. In the mild foot shock group (b), the mice were presented either a mild foot shock alone (0.3 mA for 2 s) or in conjunction with astrocytic ArchT photoactivation during conditioning (*n* = 9 and 12, respectively). In the strong foot shock group (c), the mice were presented either a strong foot shock alone (0.7 mA for 4 s) or in conjunction with astrocytic ArchT photoactivation (*n* = 14 and 10, respectively). *p* = .0227 Paired *t* test *<.025 with Holm correction for foot shock only mice, and *p* = .927 > .05 with Holm correction for foot shock+ArchT mice in (b). *p* = .0107 Paired *t* test *<.025 with Holm correction for foot shock only mice, and *p* = .967 > .05 with Holm correction for foot shock+ArchT mice in (c). Data from the same individual mice as those presented in Figures 7c,d and 8c,d. In mice conditioned with both mild and strong foot shocks, the freezing behavior had decreased after 3 weeks compared to the tests conducted the day after conditioning. (d, e) Freezing levels in the contextual fear test conducted 21 days post conditioning were normalized to that on the day after. *p* = .0265 Welch's *t* test *<.05 between mild foot shock only group and foot shock+ArchT group in (d), and *p* = .144 Welch's *t* test >.05 between strong foot shock only group and foot shock+ArchT group) in (e). While not descripted in the figure, One‐sample *t* tests compared to a hypothetical mean of 1 (perfect memory retention) was conducted. *p* = .00667 *<.025 with Holm correction for foot shock only mice, and *p* = .705 > .05 with Holm correction for foot shock+ArchT mice in (d). *p* = .0315 > .025 with Holm correction for foot shock only mice, and *p* = .825 > .05 with Holm correction for foot shock + ArchT mice in (e). The data used here were adapted from those used in (b) and (c). While memory decayed over 3 weeks in the foot shock only mice, in the astrocytic ArchT photoactivated mice, the fear memory was maintained without decay over 3 weeks.

### 3.6 ArchT suppresses memory formation of intense aversive experience

**FIGURE 10:**
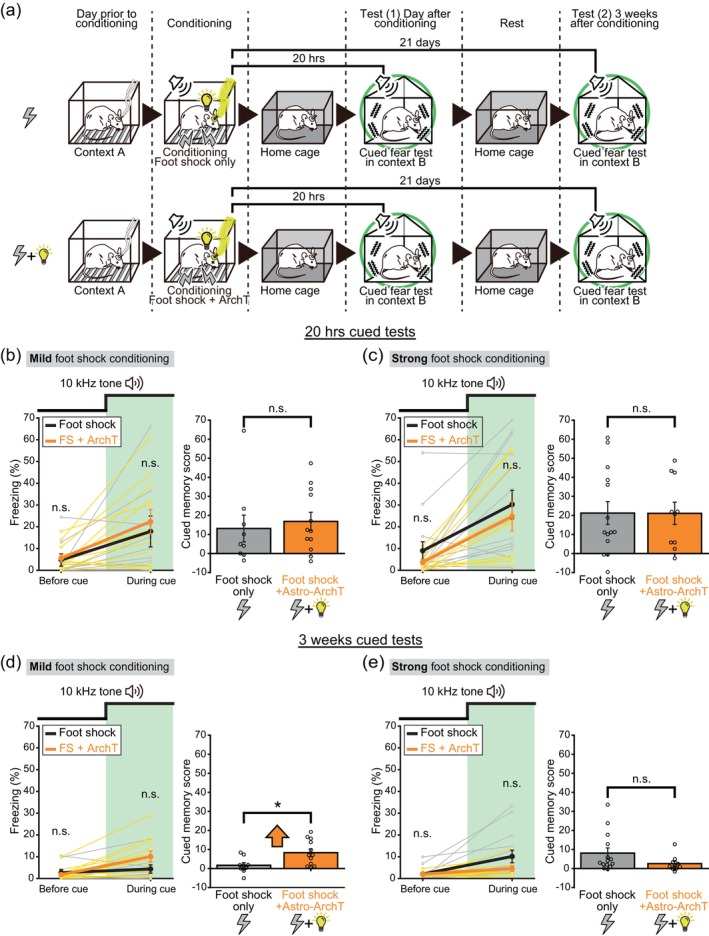
Astrocytic ArchT photoactivation inhibits remote cued fear memory decay. (a) Schematic of cued fear conditioning with and without astrocytic ArchT photoactivation during conditioning. Cued fear tests were conducted two times, once on the day following conditioning, and then again 21 days after the conditioning session. While only the cued fear tests are depicted here, contextual fear tests were also conducted with the same mice. (b) Freezing levels during cued fear tests conducted the day after the conditioning sessions are shown on the left. During conditioning, mice were either given a mild foot shock (0.3 mA for 2 s) alone or given a foot shock in conjunction with astrocytic ArchT photoactivation. Freezing levels (mean ± SEM) during the 3‐min period before (*p* = .808 Welch's *t* test >.05 with Holm correction) and during (*p* = .622 Welch's *t* test >.025 with Holm correction) the presentation of the 10 kHz tone is shown. Cued memory scores (Freezing% during tone − Freezing% before tone presentation) are shown on the right. *p* = .668 Welch's *t* test >.05. The respective sample sizes for the foot shock only group and foot shock+ArchT group are *n* = 9 and 12, respectively. The data shown here were gathered from the same individual mice as those used in Figures 7c, 8c, and 9b,d. Astrocytic ArchT photoactivation did not significantly affect freezing behavior, neither before nor during the tone presentation, nor did it influence the cued fear memory scores. (c) Freezing levels during cued fear tests conducted the day after the conditioning sessions are shown on the left. During conditioning, mice were either given a strong foot shock (0.7 mA for 4 s) alone or given a foot shock in conjunction with astrocytic ArchT photoactivation. Freezing levels (mean ± SEM) during the 3‐min period before (*p* = .236 Welch's *t* test >.025 with Holm correction) and during (*p* = .571 Welch's *t* test >.05 with Holm correction) the presentation of the 10 kHz tone is shown. Cued memory scores are shown on the right. *p* = .986 Welch's *t* test >.05. The respective sample sizes are n = 14 and 10. The data shown here were gathered from the same individual mice as those used in Figures 7d, 8d, and 9c,e. ArchT photoactivation did not significantly affect freezing behavior, neither before nor during the tone presentation, nor did it influence the cued fear memory scores. (d) Freezing levels during cued fear tests conducted 21 days after the conditioning sessions are shown on the left. During conditioning, mice were either given a mild foot shock (0.3 mA for 2 s) alone or given a foot shock in conjunction with astrocytic ArchT photoactivation. Freezing levels (mean ± SEM) during the 3‐min period before (*p* = 0.588 Welch's *t* test >.05 with Holm correction) and during (*p* = .0824 Welch's *t* test >.025 with Holm correction) the presentation of the 10 kHz tone is shown. Cued memory scores are shown on the right. *p* = .0114 Welch's *t* test *<.05. The data shown here were gathered from the same individual mice as those used in (b), Figures 7c, 8c, and 9b,d. ArchT photoactivation significantly increased the cued fear memory scores. This demonstrates the retention and absence of decay in cued fear memory. (e) Changes in freezing levels during cued fear tests conducted 21 days after the conditioning sessions are shown on the left. During conditioning, mice were either given a strong foot shock (0.7 mA for 4 s) alone or given a foot shock in conjunction with astrocytic ArchT photoactivation. Freezing levels (mean ± SEM) during the 3‐min period before (*p* = .974 Welch's *t* test >.05 with Holm correction) and during (*p* = .0985 Welch's *t* test >.025 with Holm correction) the presentation of the 10 kHz tone is shown. Cued memory scores are shown on the right. *p* = .0828 Welch's *t* test >.05. The data shown here were gathered from the same individual mice as those used in (c), Figures 7c, 8c, and 9b,d. With strong foot shock condition, ArchT photoactivation did not significantly affect freezing behavior, neither before nor during the tone presentation, nor did it influence the cued fear memory scores.

The effects of astrocytic ArchT photoactivation on fear memory were studied using the contextual and cued fear conditioning paradigm (Figure S4). Astrocytic ArchT photoactivation during conditioning (Figure 8a,b) did not affect the freezing observed during the contextual tests on the following day when the mice were conditioned with a relatively mild foot shock (Figure 8c). When a strong‐intensity foot shock alone was delivered during conditioning, higher freezing levels were observed on the following day, compared to the cases where a mild‐intensity foot shock alone was used (Figure 8c,d). Therefore, the intensity of the aversive experience correlates with the magnitude of fear memory expression on the following day. However, when astrocytic ArchT was photoactivated along with the strong foot shock, the freezing levels tested on the following day were significantly reduced (Figure 8d). In fact, regardless of whether the mice were conditioned with a mild or strong foot shock, the freezing levels observed in astrocytic ArchT photoactivated mice were very similar (Figure 8c,d). This suggests that the correlated encoding of stronger aversive experience into stronger fear memory is lost with astrocytic ArchT photoactivation. In other words, the selective filtering of intense, significant experiences into stronger memories is likely perturbed by astrocytic ArchT photoactivation during conditioning. Interestingly, this effect of astrocytic ArchT photoactivation on selective memory filtering mechanisms was not apparent in the cued fear tests (Figure 10a–c). Similar trends were observed when short‐term memory was tested 6 min after conditioning with a strong‐intensity foot shock (Figure S8).

### 3.7 Astrocytic ArchT photoactivation inhibits remote memory decay

Forgetting memories over a long period of time can be adaptive, as it is unlikely that the same aversive event will recur after a reasonable amount of time has passed. The impact of astrocytic ArchT photoactivation on 3 weeks' long memory retention was evaluated. Fear memory was tested the day after and 21 days after conditioning to assess remote memory retention (Figure 9a). In mice that recieved the foot shock alone for conditioning, the freezing levels decayed over the 3 weeks (Figure 9b,c). However, when astrocytic ArchT was photoactivated upon foot shock conditioning, the freezing levels remained constant over the 3 weeks. The freezing levels were normalized to those on the ‘Day after’ test to assess this memory decay over the 3 weeks. When the mild foot shock alone was delivered, the normalized freezing levels were significantly decayed at the ‘3 weeks after’ tests. However, when astrocytic ArchT was photoactivated, the normalized freezing levels remained constant. The memory retention rate assessed at 3 weeks was significantly different between the foot shock only condition and the foot shock plus astrocytic ArchT photoactivated condition (Figure 9d). A similar trend was also observed for the strong foot shock condition, although the difference between the foot shock only and foot shock plus astrocytic ArchT photoactivation conditions were not statistically significant (Figure 9e). These results show that astrocytic ArchT photoactivation apparently hinders the long‐term decay of fear memories over the span of three weeks. It seems important to point out that the perturbation of the astrocytic states was done solely during the conditioning session. The effect of this perturbation took three weeks to manifest in the long‐term retention of fear memory.

The impact of astrocytic ArchT photoactivation on remote memory retention was evaluated for the cued memory as well (Figure 10a). Whether astrocytic ArchT was photoactivated during the conditioning session or not, the freezing levels and cued memory scores were similar on the day following conditioning (Figure 10b,c). In the mild foot shock conditioned mice, after three weeks from the conditioning, the cued memory score was significantly higher for the astrocytic ArchT photoactivated mice compared to the foot shock only group (Figure 10d). This shows that astrocytic ArchT photoactivation during conditioning sessions results in longer retention of cued memory. In other words, long‐term decay of cued fear memory is apparently inhibited by the astrocytic ArchT photoactivation. However, this effect of astrocytic ArchT photoactivation was not apparent for the strong foot shock condition (Figure 10e). These results are consistent with those of the contextual fear tests.

### 3.8 Astrocytes acidify in response to foot shock stimuli

We have shown that disrupting natural astrocytic activity with ChR2 and ArchT can alter both acute responses to fear and long‐term fear memory formation. However, such manipulations may not fully reflect the physiological processes taking place in the brain. One possible cause for these effects could be alterations in intracellular pH due to the photoactivation of ChR2 and ArchT. ChR2 is a cation channel, and its photoactivation leads to intracellular acidification. Conversely, ArchT is an outward proton pump, and its photoactivation induces intracellular alkalization (Beppu et al., 2014). Using the pH‐sensitive fluorescence sensor E^2^GFP (Bizzarri et al., 2006) specifically expressed in astrocytes, we examined the pH changes that occur due to foot shock stimuli. These measurements were taken using fiber photometry in the amygdala (the aBLA). Astrocyte specific expression of E^2^GFP was achieved with Mlc1‐tTA::tetO‐E^2^GFP mice, and optical fibers were inserted into the amygdala for fluorescence measurements (Figure S9a).

Astrocytes became acidified when the mice received electric foot shocks, but not during control experiments with the same animals when they did not receive foot shocks (Figure S9). ChR2 and ArchT photoactivations may disrupt such natural pH changes in astrocytes. This may explain the observed alterations in fear behavior and memory formation due to these photomanipulations.

## 4 DISCUSSION

Our results demonstrate that astrocytic ChR2 photoactivation in the amygdala during fear conditioning disrupts long‐term memory formation but not short‐term memory formation. These results indicate that 6‐min short‐term memory formation is a robust process that is unperturbed even when normal astrocyte activity is strongly influenced by the artificial astrocytic ChR2 photoactivation. However, when astrocytic ChR2 is photoactivated during conditioning, the plastic changes gained by the foot shock conditioning are not handed over to 1‐h or longer‐term memory for consolidation. It is possible that influences resulting from astrocytic ChR2 photoactivation during conditioning remain in the tissue for an extended period of time and interfere with the short‐term memory to long‐term memory conversion (or memory consolidation) process, which likely takes place from 6 min to 1 h post‐conditioning. However, this is unlikely, as astrocytic ChR2 photoactivation delivered 33 min post‐conditioning failed to interfere with long‐term memory formation. Another possibility is that both short‐ and long‐term memory formation processes are triggered simultaneously during conditioning, and the two processes proceed independently in parallel (i.e., the parallel memory formation hypothesis). Short‐term memory is quickly lost between 6 min and 1 h after conditioning, and long‐term memory formation requires a certain delay of more than 6 min before it can manifest. It is possible that astrocytic ChR2 photoactivation preferentially interferes with the mechanisms related to the onset of the latter of the parallel memory formation processes. This long‐term memory formation process is apparently vulnerable to perturbations from the astrocytes only at the very moment of the aversive experience. We empirically experience that some memories are unforgettable while others fade with time. Astrocyte activity may be controlling the meta‐plasticity of the neuronal circuit. In the innate brain, astrocyte activity may spontaneously fluctuate, and the coinciding state of astrocytes at the moment of an aversive experience may determine the long‐term future fate of fear memories. The summary of findings are depicted in Figure S10.

In a previous study, Li et al. (2020) showed that continuous astrocytic ChR2 photoactivation in the rat hippocampus after conditioning can inhibit fear memory formation via an adenosine pathway. While this study does not mention the rigid short‐term memory formation process, the mechanism for long‐term memory inhibition may be similar. Intriguingly, hippocampal astrocytic ChR2 photoactivations in rats were effective even 1 h after conditioning. This could reflect the distinct roles between different brain regions in long‐term memory formation.

We also demonstrated that activating astrocytic ChR2 in the amygdala elicits complex behavioral characteristics (e.g., freezing and tail rattling) commonly observed during an aversive experience. Generally, evoking fear‐like behaviors such as freezing involves the activation of neurons with regional specificity (Applegate et al., 1983; Ciocchi et al., 2010; Johansen et al., 2010; Weingarten & White, 1978) or specified axonal projections between particular brain regions (Hwang et al., 2023; Kim et al., 2013), or it requires targeted activation of memory engrams cells (Chen et al., 2019; Liu et al., 2012). While the primary goal of this study was to manipulate astrocytes in the amygdala (particularly the aBLA), it is important to acknowledge that astrocytes in other regions may also have been influenced by the illumination via the implanted optical fibers. Although the optogenetic tools, ChR2 and ArchT, were specifically expressed in astrocytes using transgenic mice, astrocytes in other sub‐regions of the amygdala or other brain regions beyond the amygdala could also have been photoactivated. To identify the specific region related to long‐term memory consolidation and retention, targeted viral expression of the optogenetic tools would be necessary. That being said, intriguingly, we show that bulk manipulations of astrocytes in a relatively broad region can trigger intricate neuronal mechanisms, resulting in complex fear‐like behaviors. Influences of widespread astrocyte activities on precise neuronal activities could potentially mediate the effects of hormones, neuropeptides, and other broad‐acting neuroactive substances (Asrican et al., 2020; Tertil et al., 2018; Wahis et al., 2021). This interplay between astrocytes and neurons may provide a mechanism through which global brain states or systemic factors can influence detailed neuronal circuit operations.

ChR2 is a cation channel that prompts ion influx into the cell. Since ChR2 has high permeability for proton (Nagel et al., 2003), photoactivation of ChR2 leads to intracellular acidification (Beppu et al., 2014). Conversely, ArchT is an outward proton pump, and its photoactivation induces proton extrusion, resulting in intracellular alkalization (Beppu et al., 2014). It should also be noted that potential off‐target effects could arise from the use of ChR2 and ArchT in astrocytes, introducing complexities such as potassium ‘leak’ into the extracellular space (Octeau et al., 2019), and extracellular pH changes which may influence local neuronal activity (Chiang et al., 2015; Huda et al., 2013). In this study, we showed that the photoactivation of ChR2 and ArchT leads to contrasting behavioral responses; ChR2 triggers fear‐like behavioral responses, while ArchT results in a decrease in freezing. The opposing impacts of ChR2 and ArchT photoactivation on astrocytic intracellular pH may underlie this phenomenon. This implies that astrocytic intracellular pH could potentially be a determining factor of an animal's emotional state. In innate brain cells, intracellular pH can fluctuate as (a) acidic protons and alkaline bicarbonates can cross the plasma membrane via various channels and transporters, (b) acidic CO_2_ can be created by the respiration of the mitochondria and taken away with various efficiency depending on the blood flow (Sasaki et al., 2024), and (c) both acidic (e.g. lysosomes) and alkaline (e.g. mitochondria) intracellular compartments may provide the cytosol with ions which can produce pH fluctuations. In astrocytes, acidic pH in the cytosol has been shown to activate anion channels, which can release glutamate ions from the cytosol to the extracellular space, which can affect synaptic plasticity (Beppu et al., 2014, 2021; Kanaya et al., 2023), and alkaline pH has been shown to induce closure of gap junctions between astrocytes, and the extrusion of extracellular excitatory potassium can be affected by this closure (Onodera et al., 2021). It is worth noting that both ChR2 and ArchT photoactivations also trigger secondary responses in astrocytes, such as changes in intracellular calcium (Figueiredo et al., 2014; Gourine et al., 2010; Nagel et al., 2003; Shen et al., 2017) or metabolite concentration (Tang et al., 2014). Such characteristics may explain the complex effects that astrocytic manipulations can bring about, not only on fear sensation but also on fear memory formation and retention.

Previously, Adamsky et al. (2018) and Iwai et al. (2021) demonstrated that calcium elevations in hippocampal astrocytes can enhance memory formation. They achieved this through artificial manipulations of the G protein‐coupled receptor (GPCR), which induced inositol triphosphate (IP3) pathway‐mediated calcium elevations through the release from endoplasmic reticulum (ER) stores. While ChR2 does indeed induce calcium elevations, our results shed light on different pathways, including pH signaling. While there are different perspectives on the cause of ChR2 induced calcium elevations (Figueiredo et al., 2014; Yang et al., 2015), it seems that ChR2 induced calcium can trigger distinct signaling cascades compared to those triggered by GPCR pathway activations (Maltsev et al., 2023). In order to elucidate the physiological roles of astrocytes in living organisms, it is crucial to observe various aspects of astrocytic intracellular ionic and metabolic dynamics (Asano et al., 2024; Dossi et al., 2024; Ikoma, Sasaki, & Matsui, 2023; Ikoma, Takahashi, et al., 2023; Tan et al., 2024) during aversive experiences in future research.

These insights could open up potential avenues for therapeutic interventions that target astrocytes in conditions characterized by abnormal fear memory, such as post‐traumatic stress disorder. Furthermore, it would be intriguing to explore whether similar effects are seen in other types of memory beyond fear memory. Our study sheds light on the possible role of astrocytes in determining the conditions and manner in which synaptic plasticity and memory formation can occur, a phenomenon often referred to as “meta‐plasticity” (Hulme et al., 2014; Nomoto et al., 2016). In conclusion, this study emphasizes the need to incorporate astrocytes into our understanding of complex information processing in the brain.

## AUTHOR CONTRIBUTIONS

Hiroki Yamao and Ko Matsui conceived and designed the study. Hiroki Yamao performed the experiments and analyzed the data. Hiroki Yamao and Ko Matsui wrote the manuscript.

## CONFLICT OF INTEREST STATEMENT

The authors declare no conflict of interest.

## Supporting information


**Appendix S1.** Figures S1–S10.

**Table S1.** Statistical information table.

## Data Availability

The data that support the findings of this study are available from the corresponding author upon reasonable request.
