# Supplementary material for: Astrocytic determinant of the fate of long‐term memory
Source: Glia. 2024 Nov 4;73(2):309–29. doi: 10.1002/glia.24636 (PMC11662981; doi:10.1002/glia.24636)
Supplement: Supplementary file 1 — Appendix S1. Figures S1–S10. [file GLIA-73-309-s001.docx]

**GLIA**

RESEARCH ARTICLE

**Supporting Information for**

**Astrocytic determinant of the fate of long-term memory**

Hiroki Yamao^a,1^, and Ko Matsui ^b,1,2,*^

^1^Super-network Brain Physiology, Graduate School of Life Sciences, Tohoku University, Sendai 980-8577, Japan.

^2^Super-network Brain Physiology, Graduate School of Medicine, Tohoku University, Sendai 980-8577, Japan

^a^ORCID ID: https://orcid.org/0000-0001-8913-9487, Email: yamao.hiroki.r5@dc.tohoku.ac.jp

^b^ORCID ID: https://orcid.org/0000-0003-1068-9705, Email: matsui@tohoku.ac.jp

*Correspondence: matsui@tohoku.ac.jp

This document includes:

Figures S1 – S10

**Supporting Figures**

**
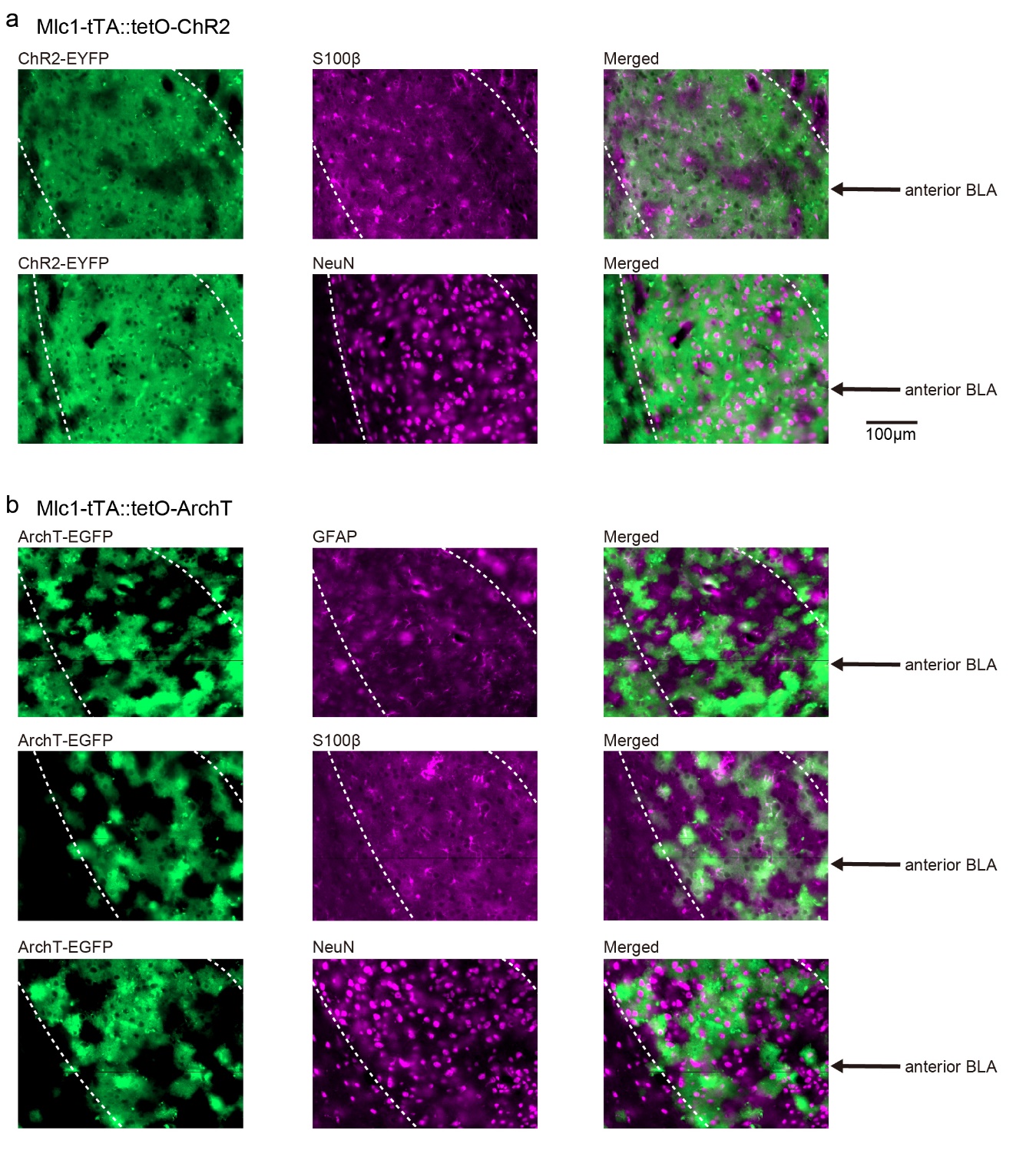
**

**FIGURE S1** Astrocyte specificity of ArchT and ChR2 expression. (a) Fluorescent images showing the distribution of ChR2(C128S)-EYFP (green) and immunohistochemistry staining for the astrocyte marker S100β (magenta) or the neuronal nucleus marker NeuN (magenta) in the anterior basolateral amygdala. ChR2 expression overlaps partially with S100β, while there is no overlap with the neuronal marker NeuN, suggesting the specificity of ArchT expression to astrocytes. (b) Fluorescent images showing the distribution of ArchT-EGFP (green) and immunohistochemistry staining for astrocyte markers GFAP or S100β (magenta) or the neuronal nucleus marker NeuN (magenta), in the anterior basolateral amygdala. ArchT expression overlaps partially with the astrocyte markers, while there is no overlap with the neuronal marker NeuN, suggesting the specificity of ArchT expression to astrocytes.

**
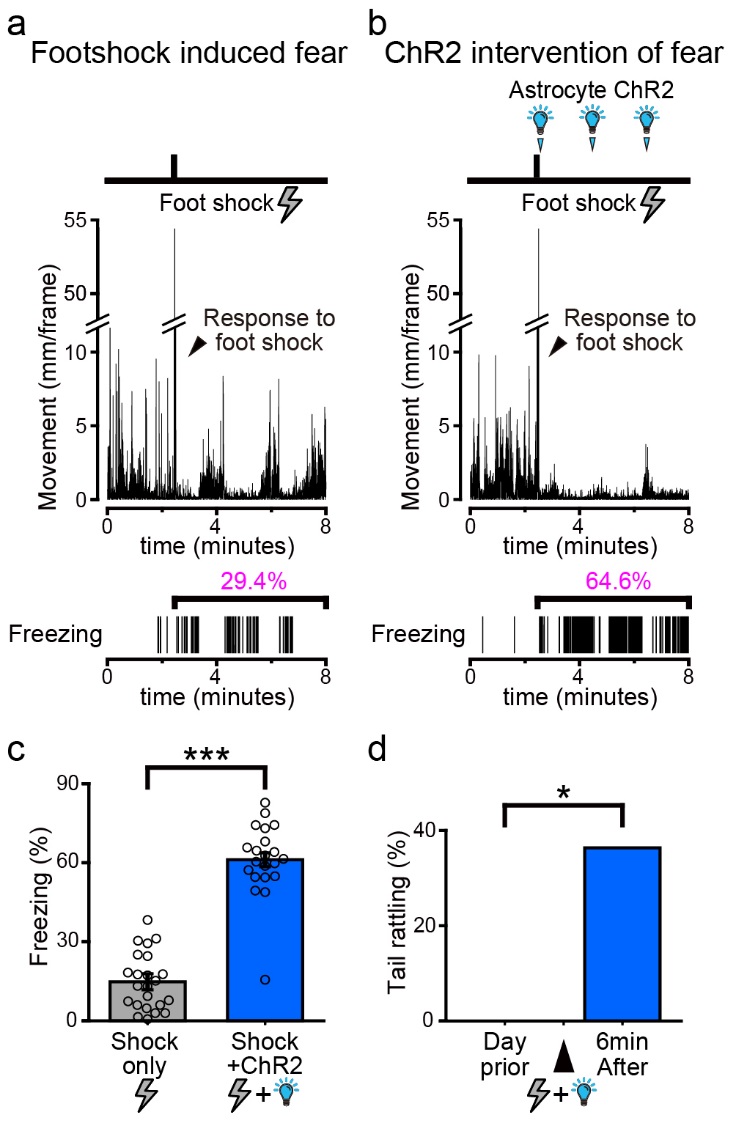
**

**FIGURE S2** Fear reaction caused by foot shock plus astrocytic ChR2 photoactivation. (a and b) Examples of mice reactions to a foot shock and astrocytic ChR2 photoactivation following a foot shock. Movement (top) and freezing (bottom) during the session. (c) Comparison of the freezing levels after a 0.7 mA, 4-second foot shock, and astrocytic ChR2 photoactivation combined with the foot shock. The freezing percentages during the 5.5-minute period following the foot shock, and during the 5.5-minute period when the mice were subjected to 3 astrocytic ChR2 photoactivations after the foot shock, are shown. Foot shock only group and foot shock + ChR2 group, p = 1.05e-14 Welch's t-test *** < 0.001, n = 23 and 21, respectively. The foot shock only group consists of the same sample set as used in Figure 2c. The data shown here were gathered from the same individual mice as those used in Figure 3e. Mice subjected to ChR2 photoactivation in conjunction with the foot shock exhibited significantly more freezing behavior. (d) Comparison of the proportion of mice that exhibited tail rattling during the 8-minute sessions on the day prior to and 6 minutes after the sessions in which they received a foot shock and astrocytic ChR2 photoactivation. Tail rattling on the day prior versus 6 minutes after the sessions, p = 0.0270 two-proportion Z-test * < 0.05, n = 11 and 11, respectively. The data shown here were gathered from the same individual mice as those used in (c) and Figure 3e. This shows that astrocytic ChR2 photoactivation does not interfere with short-term fear memory formation.

**
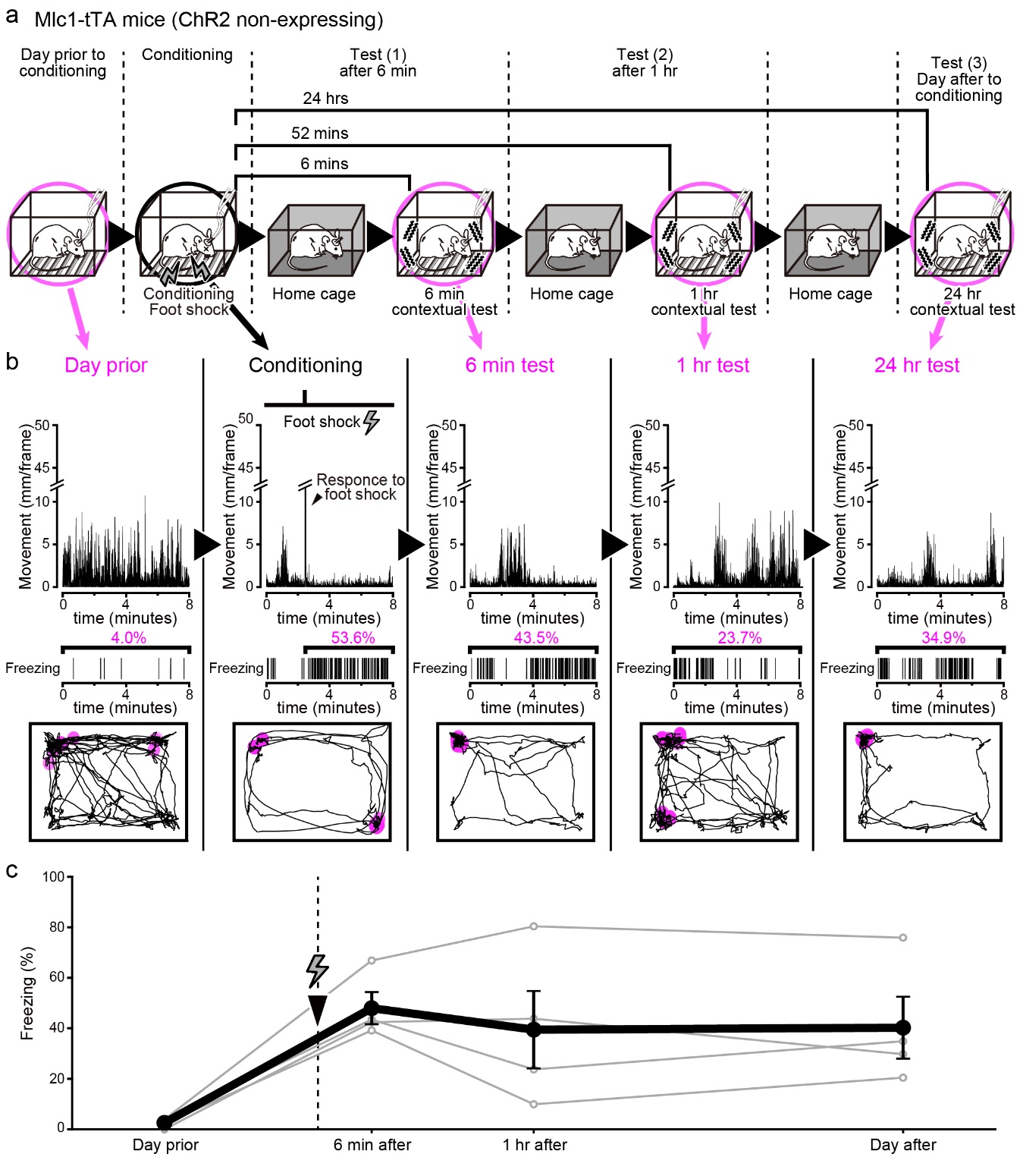
**

**FIGURE S3** Short- and long-term fear memory evaluation with foot shock conditioning. (a) Schematic of the contextual fear conditioning paradigm. (b) Examples of behaviors exhibited by a ChR2 non-expressing Mlc1-tTA mouse across five sessions. Movement (top) and freezing (middle) during the sessions are shown. Trajectories of mice during sessions (bottom) with magenta markers indicating locations where the mice displayed freezing. The test sessions were conducted the day before conditioning, 6 minutes and approximately 1 hour (52 minutes) after the conditioning session, and the day after conditioning. (c) Changes in the freezing levels (mean ± SEM) are shown for tests conducted on the previous day, 6 minutes after, approximately 1 hour after, and on the day following the foot shock conditioning sessions. The sample size was n = 4. Short-term fear memories are observable in tests conducted 6 minutes post-conditioning and are consolidated into long-term fear memories, observed during 1 hour tests and the day after tests.

**
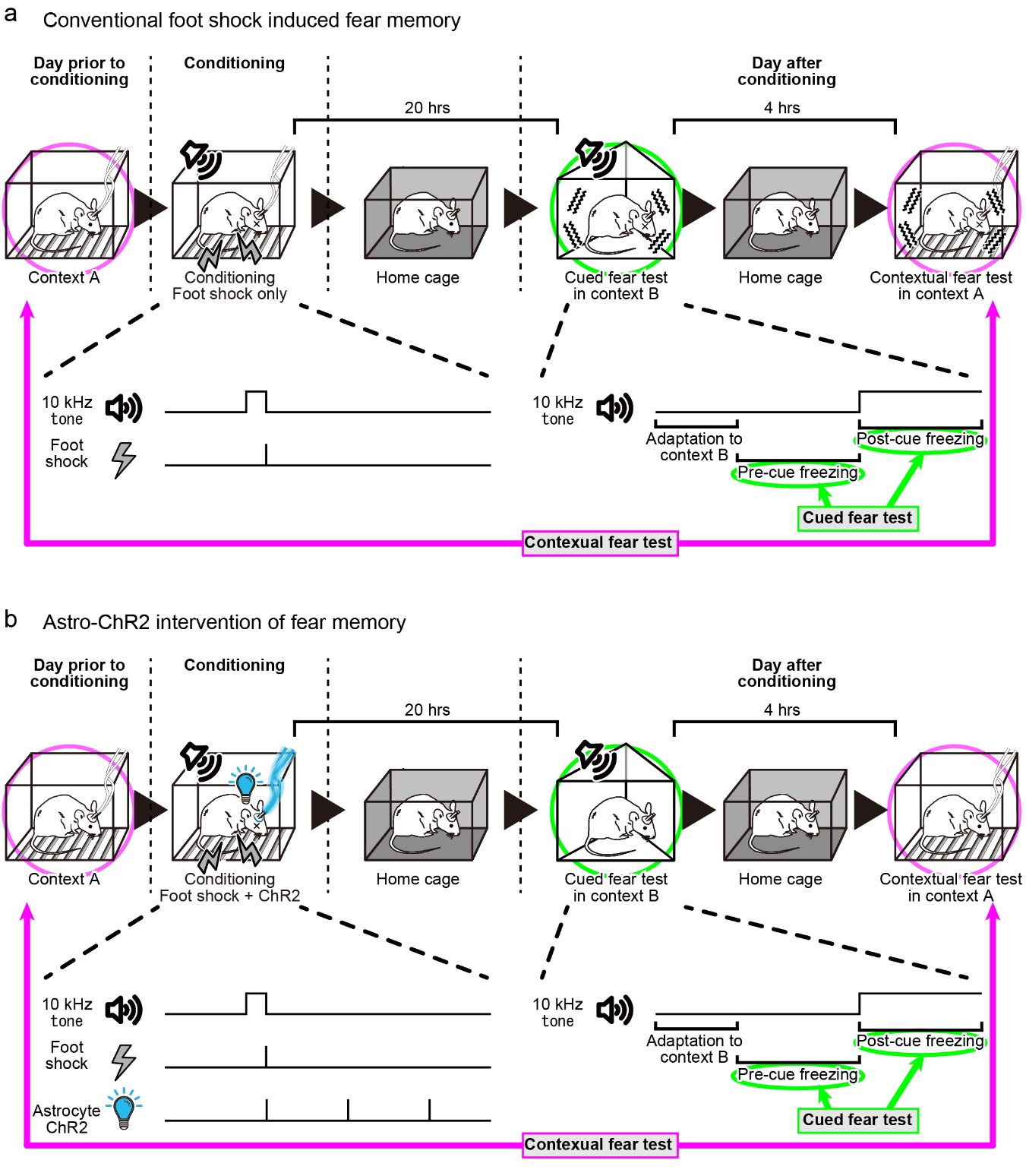
**

**FIGURE S4** Contextual and cued fear memory testing paradigm. Schematic of contextual and cued fear conditioning. During conditioning, a 30-second long tone, co-terminating with the foot shock, was presented. Mice were either subjected to or not subjected to astrocytic photoactivation following foot shocks during conditioning. When mice were subjected to photoactivation, photostimulation was administered immediately after the footshock without an interval, and an additional two times, each with a 120-second interval between them (b). Cued and contextual fear tests were conducted 20 and 24 hours after conditioning, respectively. For the cued fear test, mice were introduced into a novel context (context B). Excluding an initial adaptation period of 2 minutes, the freezing levels during a 3-minute period before and during the presentation of the 10 kHz tone were compared. For the contextual fear test, mice were reintroduced into the context in which they were conditioned. The freezing levels during the test session on the day prior to conditioning and the contextual fear test were compared to determine the increase in freezing due to contextual fear memory formation. The same principle was applied to the fear conditioning paradigm to determine the effects of both astrocytic ChR2 and ArchT photoactivation on fear memory.

**
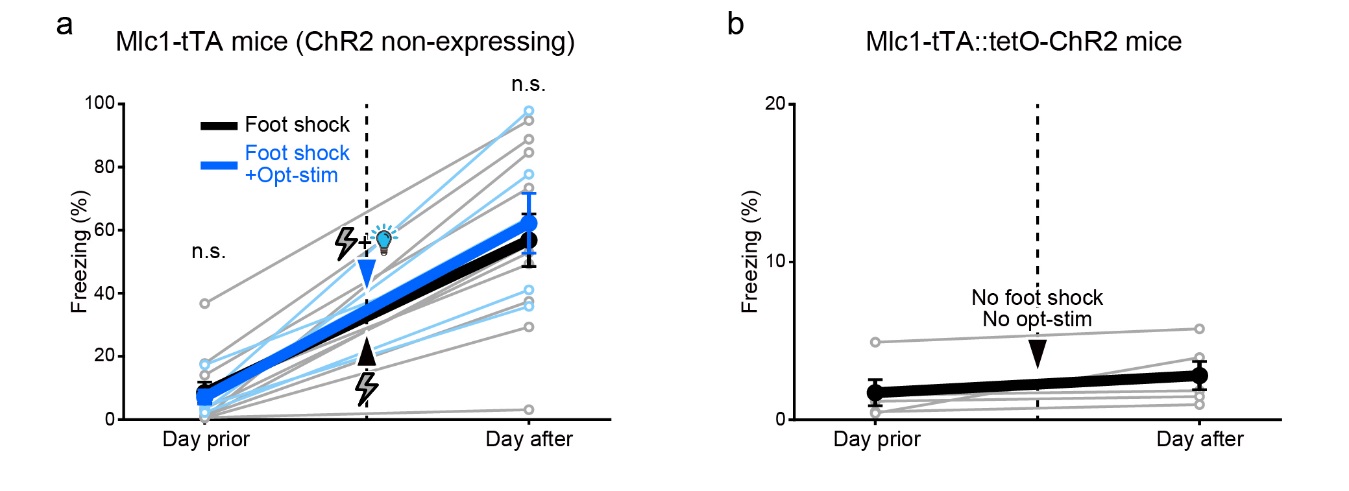
**

**FIGURE S5** Evaluation of possible artifacts on contextual memory tests. (a) Freezing levels (mean ± SEM) when tested on the previous day (p = 0.741 Welch's t-test > 0.05 with Holm correction), and on the following day (p = 0.677 Welch's t-test > 0.025 with Holm correction) of the conditioning sessions. During conditioning, ChR2 non-expressing Mlc1-tTA mice were either given a foot shock alone or given a foot shock in conjunction with photostimulation. The respective sample sizes for the foot shock only group and foot shock + Opt-stim group were n = 11 and 6, respectively. Photostimulation itself, without activation of ChR2, does not influence fear memory. (b) Freezing levels (mean ± SEM) when tested on the previous day and on the following day of the sessions. During the pseudo conditioning, sham-operated Mlc1-tTA::tetO-ChR2 mice were given neither a foot shock nor photostimulation. The sample size was n = 5. Without foot shocks or photostimulation, fear memory formation does not occur.

**
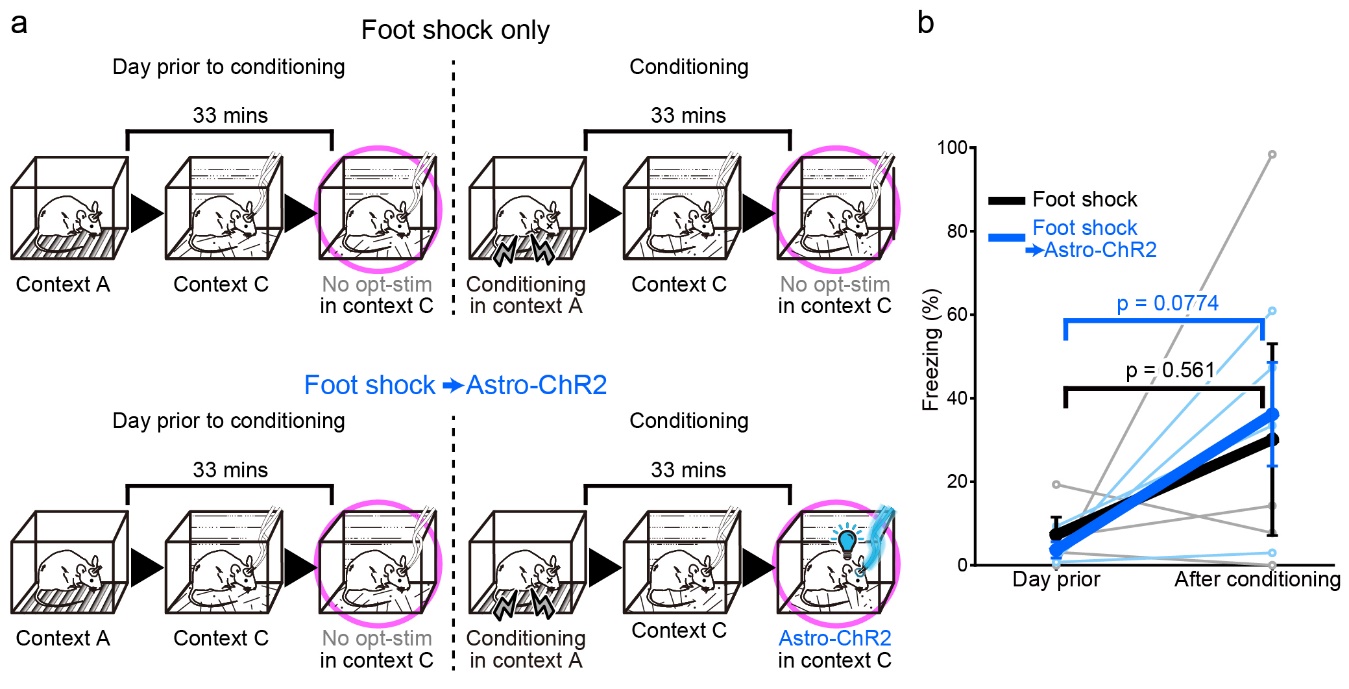
**

**FIGURE S6** Freezing induced by delayed ChR2 photoactivation. (a) Schematic of freezing behavior assessment in [context C] during the delayed photoactivation experiments. Freezing levels were assessed during the 5.5 minute period starting from 33 minutes after the fear conditioning sessions in [context A]. Freezing levels on the day prior to and 33 minutes after conditioning were compared. Mice were subjected to either delayed astrocytic ChR2 photoactivation or not during these 5.5 minutes. (b) Freezing levels tested on the day prior and 33 minutes after the conditioning sessions. Foot shock only group (p = 0.561 Welch's t-test) and foot shock + delayed ChR2 group (p = 0.0774 Welch's t-test), n = 4 and 4, respectively -Data from the same individual mice presented in Figure 4b. Freezing levels increased due to ChR2 photoactivation in [context C]. Freezing levels also increased in the foot shock only group compared to the day prior. However, this was only due to the large increase in the freezing level of a single individual, reflected by the large p-value. The particular individual seemed to be sleeping, although undeterminable with the lack of methods such as EEG recordings. This likely caused a false detection of freezing behavior due to the lack of motion.


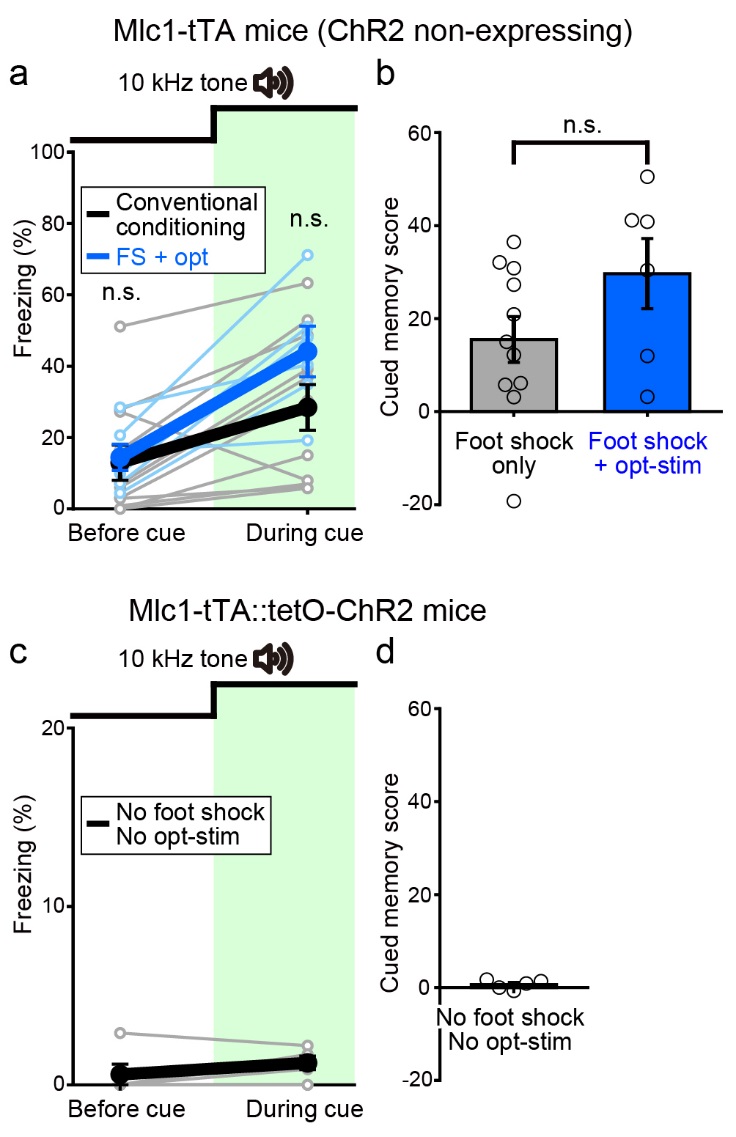


**FIGURE S7** Evaluation of possible artifacts on cued fear memory tests. (a) Freezing levels during cued fear tests conducted the day after the conditioning sessions. During conditioning, ChR2 non-expressing Mlc1-tTA mice were either given a foot shock alone or given a foot shock in conjunction with photostimulation. Freezing levels (mean ± SEM) during the 3-minute period before (p = 0.812 Welch's t-test > 0.05 with Holm correction) and during (p = 0.126 Welch's t-test > 0.025 with Holm correction) the presentation of the 10 kHz tone is shown. The sample sizes for the foot shock only group and foot shock + Opt-stim group are n = 11 and 6, respectively. The data shown here were gathered from the same individual mice as those used in Figure S5a. Photostimulation itself, without activation of ChR2, did not significantly affect freezing, neither before nor during the tone presentation. (b) Cued memory scores (Freezing % during tone - Freezing % before tone presentation) are shown. The data is taken from the same sample set as used in (a). p = 0.149 Welch's t-test > 0.05. Photostimulation itself without activation of ChR2 did not significantly affect the cued memory scores, indicating that it has no impact on cued fear memory. (c) Freezing levels during cued fear tests conducted the day after the conditioning sessions. During conditioning, sham-operated Mlc1-tTA::tetO-ChR2 mice were given neither foot shocks nor photostimulations. Freezing levels (mean ± SEM) during the 3-minute period before and during the presentation of the 10 kHz tone is shown. The sample size is n = 5. The data shown here were gathered from the same individual mice as those used in Figure S5b. Mice that were given neither foot shocks nor photostimulations exhibited negligible freezing behavior both before and during tone presentation. (d) Cued memory scores (Freezing % during tone - Freezing % before tone presentation) are shown. The data is taken from the same sample set as used in (c). Mice that were given neither foot shocks nor photostimulations had negligible cued memory scores, indicating that cued fear memory was not formed.


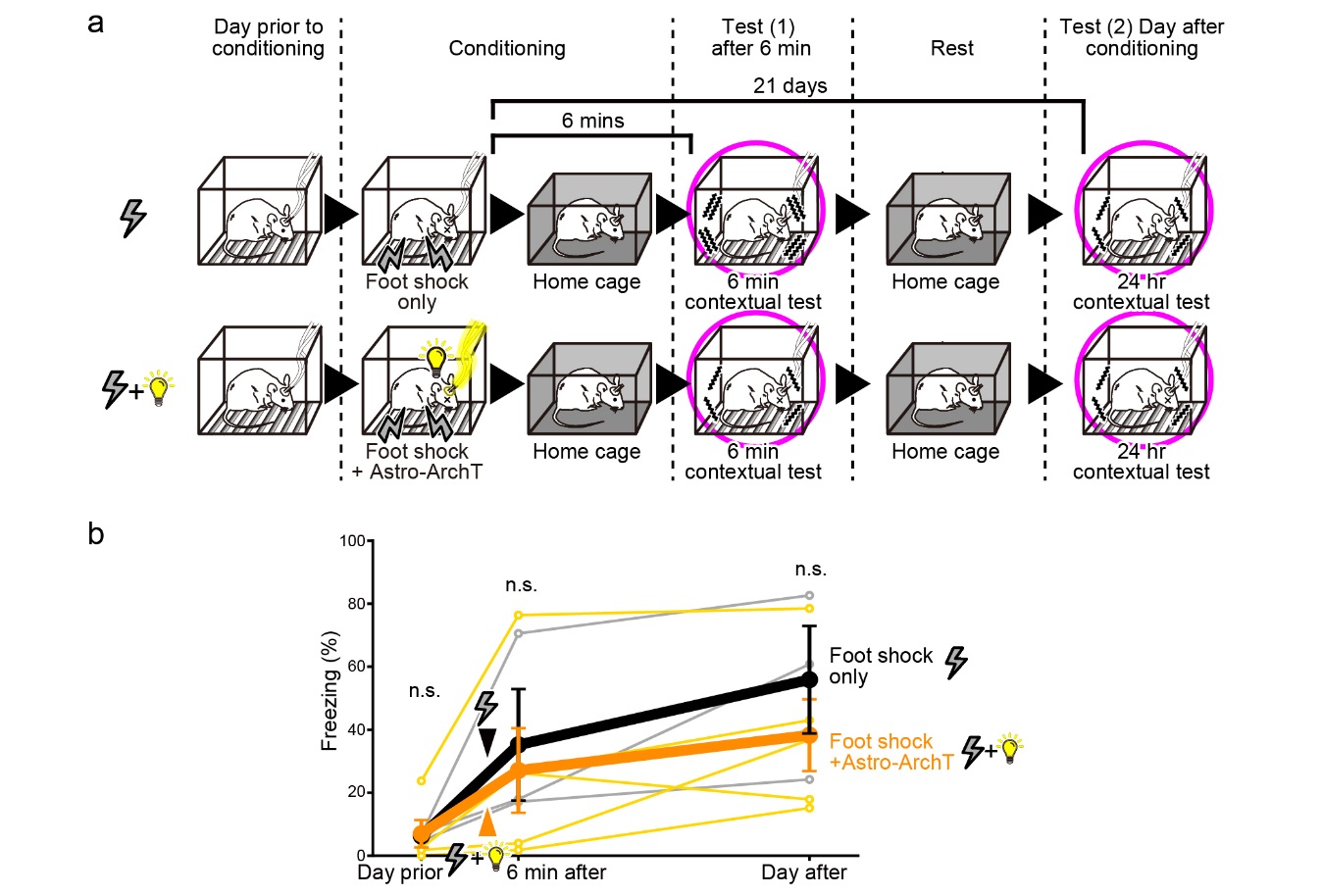


**FIGURE S8** Effect of astrocytic ArchT photoactivation on short-term memory. (a) Schematic of short- and long-term tests following conditioning with and without ArchT photoactivation. (b) Freezing levels (mean ± SEM) when tested on the previous day (p = 0.551 Welch's t-test), 6 minutes after (p = 0.732 Welch's t-test), and on the following day (p = 0.371 Welch's t-test) of the conditioning sessions. During conditioning, foot shock alone (n = 3) or foot shock in conjunction with astrocytic ArchT photoactivation (n = 5) was delivered. Astrocytic ArchT photoactivated mice showed less freezing both 6 minutes after and on the following day.


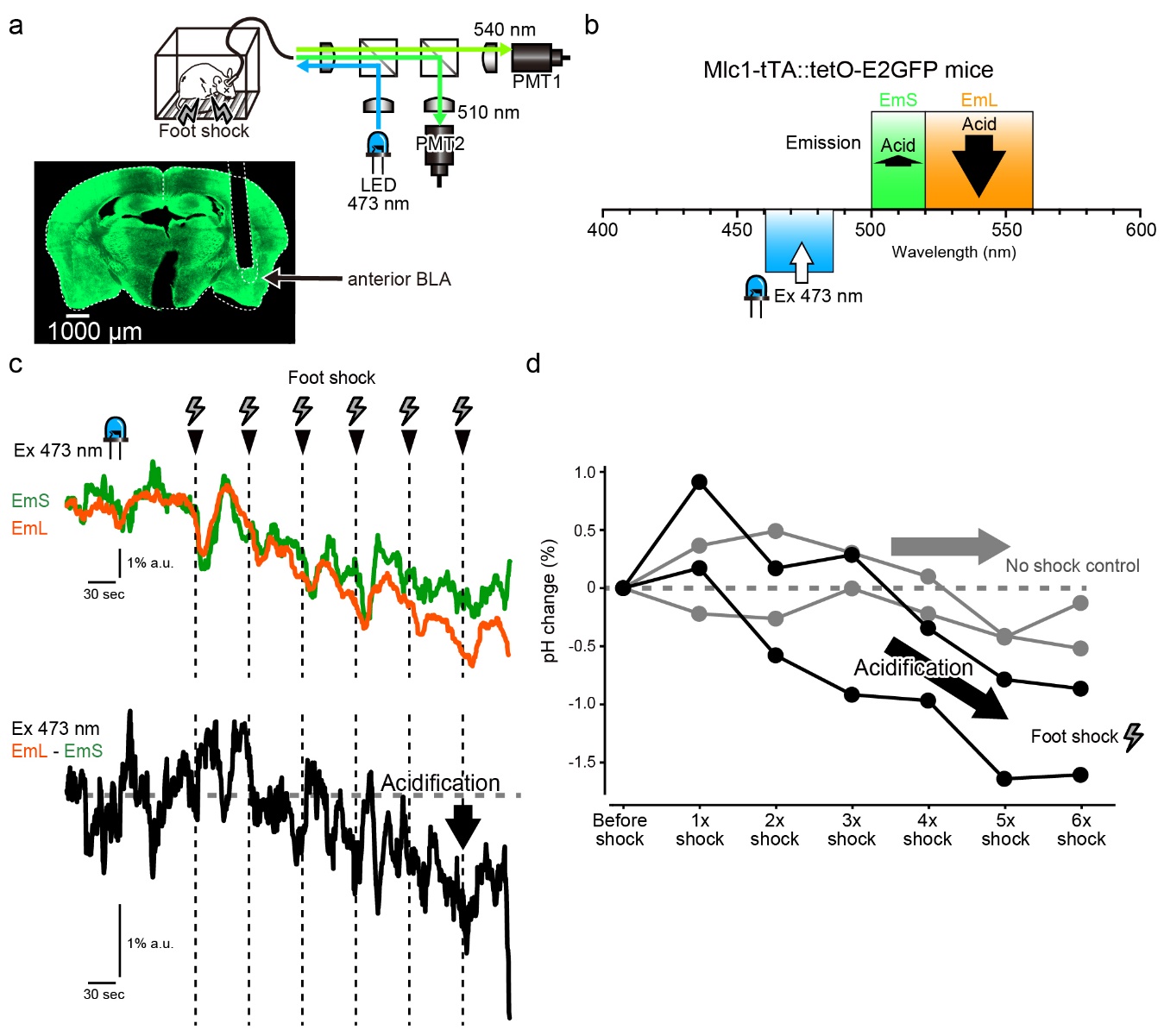


**FIGURE S9** Astrocytes acidify in response to foot shock stimuli. (a) Schematic of fiber photometry recording. Mice expressing the pH sensor Lck-E^2^GFP, specifically in astrocytes, were used. (b) 473 nm light was used for excitation. The fluorescent signals measured at ∼540 nm (EmL) are expected to decrease with acidification. Meanwhile, the signal measured at ∼510 nm (EmL), near the isosbestic point, is expected to remain relatively constant regarding pH changes. Thus, the difference between the two signals (EmL – EmS) should result in an estimate of pH dynamics with negative deflections that reflect the intracellular acidification of astrocytes. (c) Traces of EmL (orange) and EmS (green) signals (top) and the difference between the two signals EmL – EmS (bottom) during the time when the mice received electric foot shocks. The difference between the two signals increased after the mice received the foot shocks, indicating the intracellular acidification of astrocytes in response to the stimuli. All traces shown are averages from two animals with a median filter applied (window size = 21). (d) Transition of the average difference between the two signals, EmL – EmS, during the 60-second period after each foot shock, normalized to the 138-second period before the mice received any foot shocks (n = 2). Acidification was more pronounced compared to the control condition on the previous day when the mice did not receive foot shocks, as shown in grey.

**
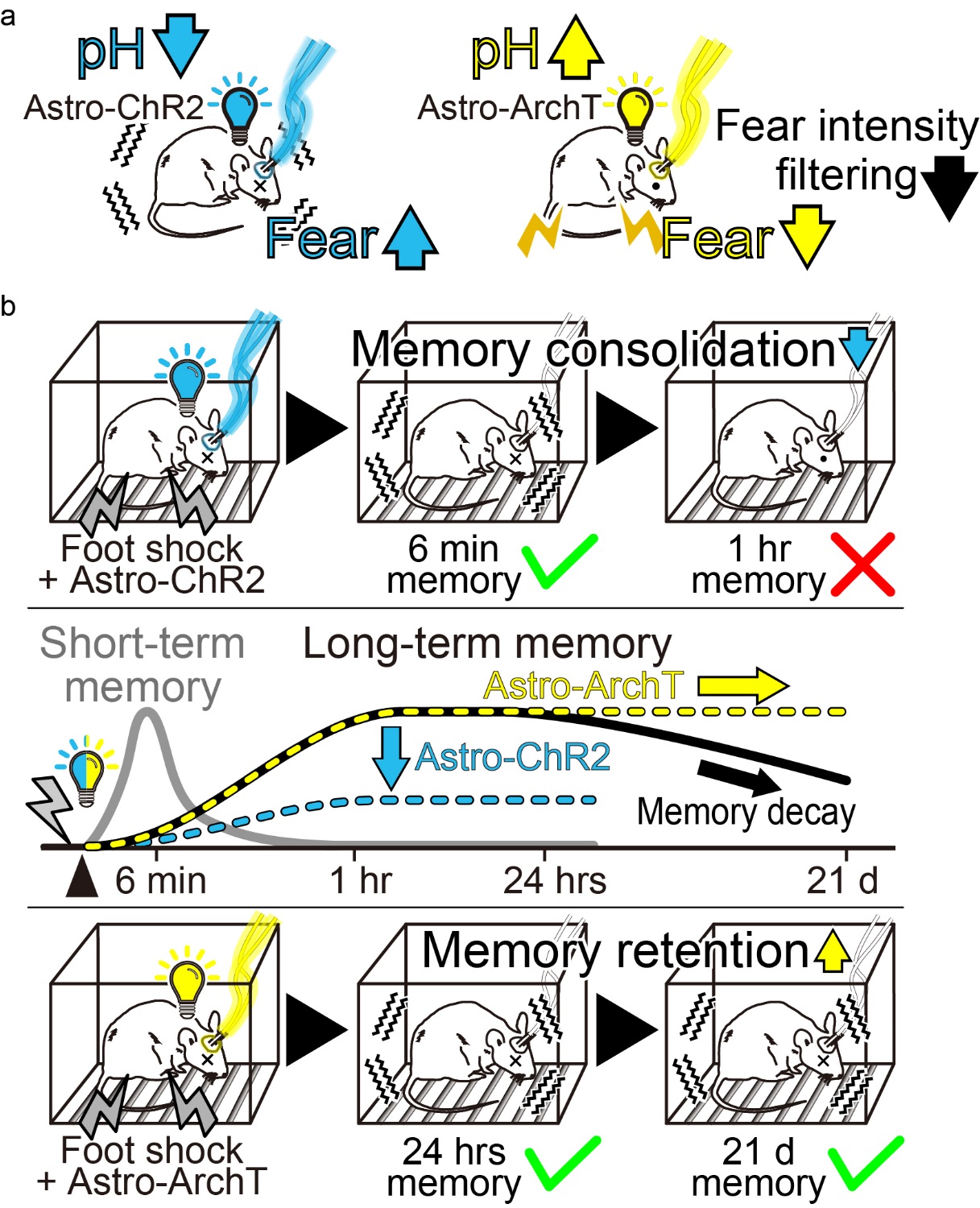
**

**FIGURE S10** Schematics of experimental findings: Astrocytic regulation of fear perception and memory formation. (a) Astrocytic ChR2 activation causes a fear-like state, while ArchT reduces fear. The ArchT reduction of fear was only observed for strong, intense foot shocks, indicating the disruption of the fear intensity filtering function. (b) Astrocytic ChR2 during conditioning suppresses long-term but not short-term memory formation. Astrocytic ArchT during conditioning results in inhibited remote memory decay over 3 weeks. Long-term memory formation is initiated at the very moment of an experience, in parallel to short-term memory formation, and is dependent on the coinciding astrocytic state.
